# Gallic Acid Based Polymers for Food Preservation: A Review

**DOI:** 10.1021/acsomega.4c05642

**Published:** 2024-08-26

**Authors:** Gayathri Gangadharan, Sonali Gupta, Manas Laxman Kudipady, Yashoda Malgar Puttaiahgowda

**Affiliations:** † Department of Chemistry, Manipal Institute of Technology, Manipal Academy of Higher Education, Manipal, Karnataka, India 576104; ‡ Department of Information and Communication Technology, Manipal Institute of Technology, Manipal Academy of Higher Education, Manipal, Karnataka, India 576104

## Abstract

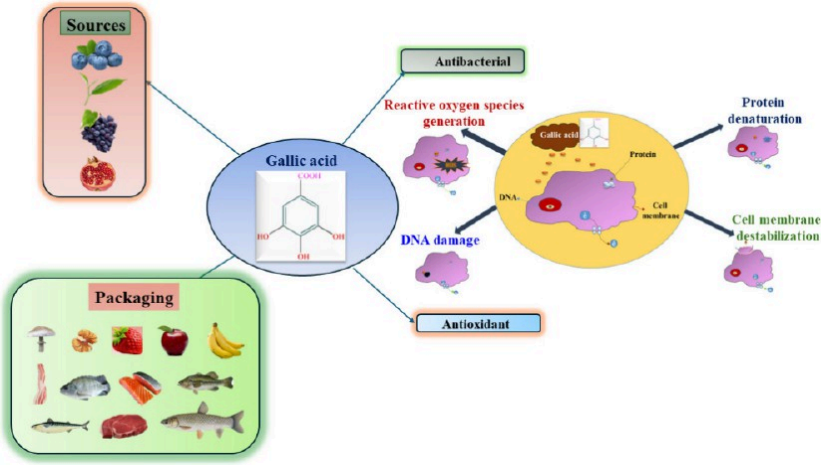

The extensive usage of nonbiodegradable plastic materials for food packaging is a major environmental concern. To address this, researchers focus on developing biocompatible and biodegradable food packaging from natural biopolymers, such as polysaccharides, proteins, and polyesters. These biopolymer-based packaging materials extend the shelf life of food due to their inherent antimicrobial and antioxidant properties. An important additive that enhances these beneficial effects is gallic acid (GA), a naturally occurring phenolic compound. GA exhibits potent antioxidant activity by scavenging free radicals and excellent antimicrobial activity against a wide range of bacteria by disrupting cell membranes. These gallic acid based active packaging solutions have demonstrated remarkable abilities to inhibit lipid oxidation, enzymatic browning, and microbial contamination and even retard the ripening processes in mushrooms, walnuts, strawberries, fresh-cut apples, bananas, fish, pork, and beef. This review focuses on the antioxidant, antibacterial, and food preservation capabilities of GA-incorporated biodegradable food packaging materials as an eco-friendly alternative to conventional plastic packaging.

## 1 Introduction

The usage of plastics is a great concern in our day-to-day lives. The present era, which is focused on sustainable development, points out the importance of decreasing the usage of conventional plastics and shifting focus to using ones which are biocompatible and that help in the overall betterment of living standards. Among plastics, food packaging is a major threat to Mother Earth, and the research now focuses on developing the ones that reduce wastage.^1^ The usage of nonbiodegradable food packaging plastic materials is nowadays being replaced by biodegradable ones due to the extensive damage they are causing to the ecosystem.^2^ Recently, the food packaging industry has been more concerned with extending the food product’s shelf life, quality, and safety by physical, chemical, and biological factors. In fabricating an active packaging material, including an active substance in the framework that acts accordingly and controlled release of it are necessary.^3^ Natural biopolymers are now in use in the production of biodegradable food packaging.

Polysaccharides, lipids, and proteins are usually incorporated. The most important point is that these synthesized compounds can improve the shelf lives of food products similar to or even better than traditional food packaging.^4^ This is attributed to the inherent antibacterial and antioxidant activities within biopolymers.^5^ Starch, cellulose, chitosan, and pectin are commonly used polysaccharides in the synthesis. The biodegradability, nontoxicity, and also biocompatibility of these molecules highlight them as important constituents in this kind of food packaging.^6^ Proteins like gelatin, soy protein, whey protein, zein, wheat gluten, casein, etc., are also commonly used. Also biopolyesters like poly(lactic acid) (PLA) and poly(vinyl alcohol) (PVA) form important constituents in the food packaging industry.^7^ In food packaging films and coatings, additives are added to improve the properties. Plant-derived phenolic acids impart antioxidant and antimicrobial properties, which makes them important.^8^ Ferulic acid, gallic acid (GA), salicylic acid, chlorogenic acid, caffeic acid, and *p*-hydroxy benzoic acid (PHA) are the phenolic acids that are incorporated as additives. Gallic acid is particularly important due to its inherent antimicrobial and antioxidant activities.

Gallic acid (GA; 3,4,5-trihydroxy benzoic acid) is a naturally occurring triphenolic compound with a low molecular weight and has been proposed in numerous studies to exhibit potential antioxidant properties, and it also acts as a cross-linker. GA is normally found in tea leaves, berries, grapes, pomegranates, capers, and wines.^9,10^ It can also be derived from chestnuts, oak trees, and plenty of other sources.^11−13^ GA, a breakdown product of propyl gallate, exhibits strong antioxidant, antibacterial, anti-inflammatory, antidiabetic, antiobesity, antimutagenic, and anticancer properties.^8,14,15^ GA can precipitate proteins and form complexes with toxic metal ions, thereby reducing their bioavailability in the environment.^16^ Its ability to alter the mechanical properties of natural biopolymers, functioning as a natural phenolic cross-linker or plasticizer, has resulted in its utilization as a material for food packaging. Due to its excellent benefits, it is commonly used as a food additive and also used in the pharmaceutical and cosmetic industries.^17^ In the food industry, it plays a role as an antioxidant, an antimicrobial, an oil stabilizer, a food wrap, and a food processing stabilizer. Also, according to the U.S. FDA, GA is recognized as a safe compound.^18^ Potential toxicity due to GA migration has been evaluated for consumer safety. At a dose of 3000 mg/kg, GA resulted in a lower survival rate of *Zophobas morio* larvae (40%). Additionally, a concentration of 2191.51 mg/kg of GA produced an acute lethal concentration (ALC50) at 48 h.^19^ Supplementing 5% GA in F344 mice resulted in lower body weight gain compared to mice not consuming GA. It was also observed that administering 0.6 and 5% GA to male and female mice, respectively, reduced hemoglobin levels, hematocrit, and red blood cell counts, while increasing reticulocyte counts.^20^ In another study, it was observed that feeding albino mice 900 mg/kg/day of GA for 28 days resulted in no significant changes in behavior or morphology.^21^ It is observed that the toxicity of GA depends on the dose and test model conditions.

There have been numerous reports on the use of GA as a filler in various bulk processing methods, including the extrusion, lamination, and coextrusion of polymers such as LDPE and PLA. In an alkaline environment, GA oxidizes, forming hydrogen peroxide, quinones, and semiquinones, making it an effective oxygen scavenger.^22^ Friedman et al. observed that GA is unstable under high pH and it gets transformed into various quinone intermediates.^24^ GA acts as an antioxidant by scavenging the available free radicals. Also, it reduces and inhibits the generation of free radicals and also quenches the oxidation of cellular oxidizable substrates. Due to the strong reducing ability and weak metal chelating ability of GA in low concentrations, it acts as a prooxidant (producing ROS), and due to its scavenging ability at higher concentrations, it acts as an antioxidant.^25^ It was reported that, in the light-induced oxidation of GA, proton-coupled electron transfer to dissolved oxygen generates hydrogen peroxide. The subsequent photolysis generates hydroxyl radicals. These ROS directly attack cellular targets, producing higher intercellular oxidative stress compared to individual treatments.^26^ Excellent antimicrobial activity is also exhibited by GA, which serves as an important parameter similar to antioxidant activity in the case of food packaging. To a wide range of bacteria, GA exhibited antibacterial activity. The antimicrobial effects of phenolic compounds can engage in several mechanisms, including destabilizing and permeabilizing the cell membrane and inhibiting enzymes via oxidized products, potentially by interacting with sulfhydryl groups or through less specific interactions with proteins, such as generating reactive quinones that can interact with amino acids and proteins. Additionally, phenols can cease the synthesis of nucleic acids in both Gram-negative and Gram-positive bacteria. In the case of GA, it can alter the hydrophobicity of bacteria. In an interaction with GA the electron acceptor ability of Gram-positive bacteria increases, whereas it decreases for Gram-negative bacteria. Due to anionic groups like carboxyl and phosphate, the bacterial cell membrane is negatively charged. After the exposure of bacterial cells to GA, its zeta potential becomes less negative. It was mainly seen in Gram-negative rather than in Gram-positive bacteria, which may be the reason for the higher susceptibility of Gram-negative bacteria. Because of the partially lipophilic nature, it is hypothesized that phenolic acids traverse the cell membrane through passive diffusion while in their undissociated state. This process disrupts the cell membrane structure and potentially leads to cytoplasmic acidification and protein denaturation. Localized hyperacidification disrupting the cell membrane is a potential mechanism that may elucidate the antimicrobial activity of phenolic acids against microorganisms.^27^ This review focuses on the antibacterial activity, antioxidant activity, and also food preservation ability of GA-incorporated food packaging films and coatings, and how the shelf lives of fruits and vegetables (section 3) and meat and fish products (section 4) have been enhanced is discussed.

## 2 Preparation of Packaging Material Containing Gallic Acid

For synthesizing phenolic-*g*-chitosans, three methods are mainly used: free radical initiation, carbodiimide coupling, and enzyme catalysis. Redox initiators such as ascorbic acid and hydrogen peroxide are utilized in initiating the free radical reaction. This method is cheap, requires moderate reaction conditions, and has low toxicity. The carbodiimide coupling reaction requires substantial quantities of chemical cross-linking reagents, which are both costly and environmentally harmful. These reagents may cause toxic or other adverse effects on the human body when grafted products are utilized in the food and pharmaceutical industries. Though enzyme catalysis is a green method, during the grafting process the phenolic compound gets oxidized, and thereby the antioxidation ability decreases.^28^ Thus, prepared phenolic-*g*-chitosan is made into a film-forming solution using the appropriate solvent and films are prepared using the solution casting technique.^29^ GA becomes unstable, and it degrades on heat, light, or oxygen exposure. Due to this reason for adopting encapsulation techniques like ionotropic gelation, coacervation, spray drying, micro- and nanoemulsions, and electrohydrodynamic process, the GA moiety gets shielded from moisture and light and thereby preserves its stability and efficacy, which helps in its use in the industrial sector.^30^ Spray drying is normally employed. However, the high spray drying temperature may lead to the structural destruction of GA and result in the loss of functional characteristics. In the case of food preservation, when GA is incorporated for its encapsulation, electrospinning is normally used. It is cost-effective and does not necessitate heating during the electrospinning process, and it produces a fiber mat with a high surface-to-volume ratio and high porosity. Due to this, the excellent properties of GA can be made available.^31^ For the preparation of active film strips containing GA, melt compounding followed by compression using a hydraulic presser was carried out. The active films synthesized were excellent oxygen scavengers.^32^ Plasma modification technology has been utilized in synthesizing PE film coated with GA and CS. This technique helps in modifying the material’s surface and also helps in incorporating new functional groups.^33^ Promsorn et al. utilized cast film extrusion for the preparation of GA loaded films on a large scale. The melted polymer resin was extruded with a twin screw extruder using this technique. The films thus produced had an oxygen scavenging ability.^34^

## 3 Reported Literature on Fruits and Vegetables

**Figure 1:**
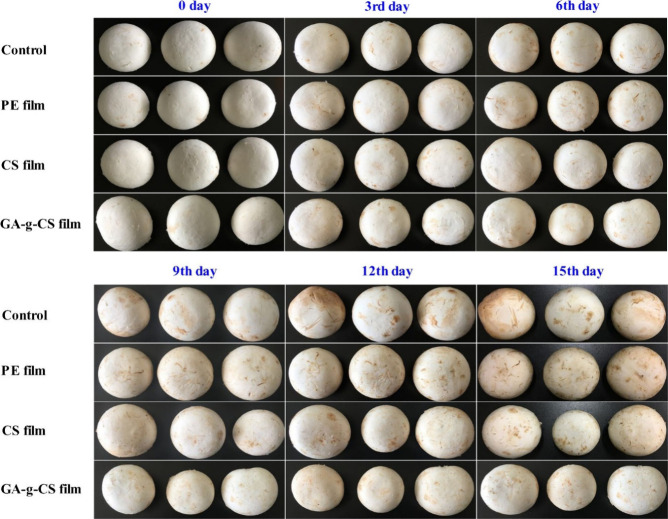
Impact of various packagings on the physical appearance of mushrooms stored at 4 ± 1 °C over different periods. Adapted with permission from ref (29). Copyright 2019 Elsevier.

In 2019 Liu and co-workers synthesized gallic acid grafted chitosan films (GA-*g*-CS) for the preservation of white button mushroom (*Agaricus bisporus*) at 4 °C.^29^ The same research group in 2017 synthesized and analyzed the changes in the mechanical, antioxidant, and physical properties of chitosan films by incorporating various hydroxybenzoic acids like gallic acid (GA), protocatechuic acid, vanillic acid, gentisic acid, and syringic acid in the carbodiimide mediated coupling reaction. Among these, GA-*g*-CS has showcased higher tensile strength, the highest DPPH radical scavenging ability, and more surface area which may be attributed to the highest grafting ratio, lowest water vapor permeability, and highest UV barrier properties. In this study, mushrooms were packed in polyethylene film (PE), chitosan film (CS), and GA-*g*-CS film and one was kept without packing for analysis (Figure 1). On analyzing the weight loss, mushrooms packed in GA-*g*-CS film had a higher weight loss than those packed in PE, but it was of an acceptable limit.^35^ On analyzing the respiration rate, it was observed that mushrooms packaged using GA-*g*-CS film were only 89.7 and 80.2% of those observed in mushrooms packaged with CS and PE films, respectively. GA can scavenge the O_2_ that penetrates through the film and also decrease residual O_2_ within the film; due to this reason the O_2_ concentration that is consumed by mushrooms during storage was reduced more in GA-*g*-CS films by day 15.^36^ Similarly, the CO_2_ concentration was lowest for GA-*g*-CS films; for this reason, the firmness of mushrooms was maintained. Lipid peroxidation happens in stored mushrooms, and malondialdehyde (MDA) is one of the byproducts which is considered a prominent biomarker for this process. GA-*g*-CS exhibited the lowest MDA content, which may be attributed to the protective effect of GA-*g*-CS on cell membranes against damage caused by free radicals.^37,38^ On mushroom ripening, antioxidant enzymes like superoxide dismutase (SOD) and catalase (CAT) are vital for antioxidant defense. SOD facilitates the dismutation of O_2_^•–^ into H_2_O_2_, which is subsequently eliminated by CAT.^39^ Mushrooms in GA-*g*-CS film exhibited higher SOD activity than those wrapped in PE film. CAT inhibits oxidative damage due to H_2_O_2_ in plant cells.^38^ Mushrooms packaged in GA-*g*-CS film showed higher CAT activity compared with those packed in PE film. The ROS level seems to be decreased due to GA-*g*-CS, thereby enhancing the antioxidant defense mechanism in mushrooms. Polyphenol oxidase (PPO) is a pivotal enzyme contributing to enzymatic browning in mushrooms. On analyzing the PPO content, it was clear that mushrooms packed in GA-*g*-CS film had lower brown pigmentation. From the observations made it was clear that GA-*g*-CS film had an excellent ability to preserve mushrooms.^29^

**Figure 2:**
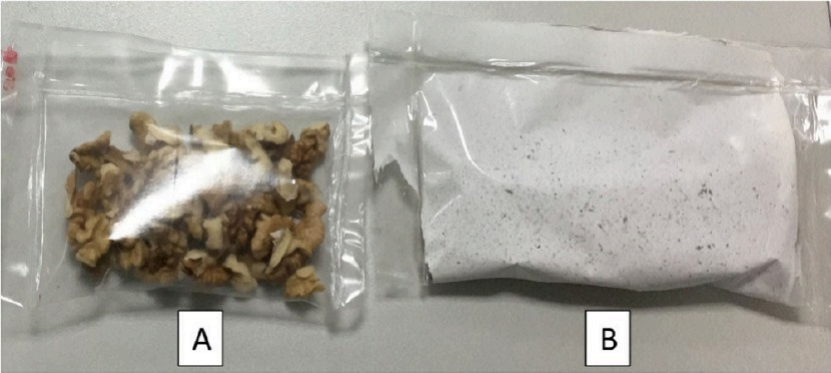
Packaged walnuts: (A) control and (B) packaged with GA-loaded nanofibers. Adapted with permission from ref (1). Copyright 2019 Elsevier.

Aydogdu et al. in 2019^1^ synthesized poly(ethylene oxide) (PEO)/lentil flour/gallic acid nanofibers for the preservation of walnuts using an electrospinning technique (Figure 2). PEO (3.5% w/v) solution and lentil flour (5.25 w/v) were mixed in a high-speed homogenizer. Two different solutions of this combination were prepared, where one maintained pH 1 and the other maintained pH 10. GA (0.1 g/mL) dissolved in 80% ethanol was introduced into these solutions. The ones without GA were considered as controls. The electrospinning technique which operates at room temperature is an advantage in preparing the fibers because GA is sensitive to higher temperature, light, and oxygen.^40^ Due to the presence of a higher amount of GA in pH 1, excellent antioxidant ability was exhibited by nanofibers at pH 1 rather than at pH 10. TGA studies revealed that the *T*_onset_ of the first degradation of nanofibers at pH 10 was higher, indicating their increased thermal stability. Also, a decrease in *T*_onset_ in both pH 1 and pH 10 nanofibers was observed by the addition of GA, and this may be attributed to the decrease in several protein–protein bonds.^41^ Walnut—an important source of polyunsaturated fatty acid (PUFA) whose intake reduces blood pressure and cholesterol—was taken for a food packaging study. PUFA undergoes easy oxidation, which thereby reduces the shelf life of walnuts. Though nanofibers at pH 1 exhibited greater antioxidant activity, nanofibers at pH 10 were considered for packaging studies. On examination of the morphology of fibers of pH 1, it was revealed that fibers adhered to one another in certain areas, likely due to solvent evaporation. Nanofibers were electrospun onto PLA sheets, and walnuts were wrapped with the nanofibers facing inward for food contact. A peroxide value of of 1.3 mequiv of O_2_/kg of walnut oil was shown by walnuts wrapped in nanofiber-coated sheets and 2.3 mequiv of O_2_/kg of walnut oil was exhibited by the control, indicating high oxidation in the control.^42^ The TOTOX value, which indicates the overall stability of oxidation, was considerably more for the control. The TBARS value did not show any considerable difference between the two samples under consideration. Given their biodegradability and antioxidant properties, lentil flour nanofibers loaded with gallic acid have significant potential to improve the oxidative stability of food products.^1^

**Figure 3:**
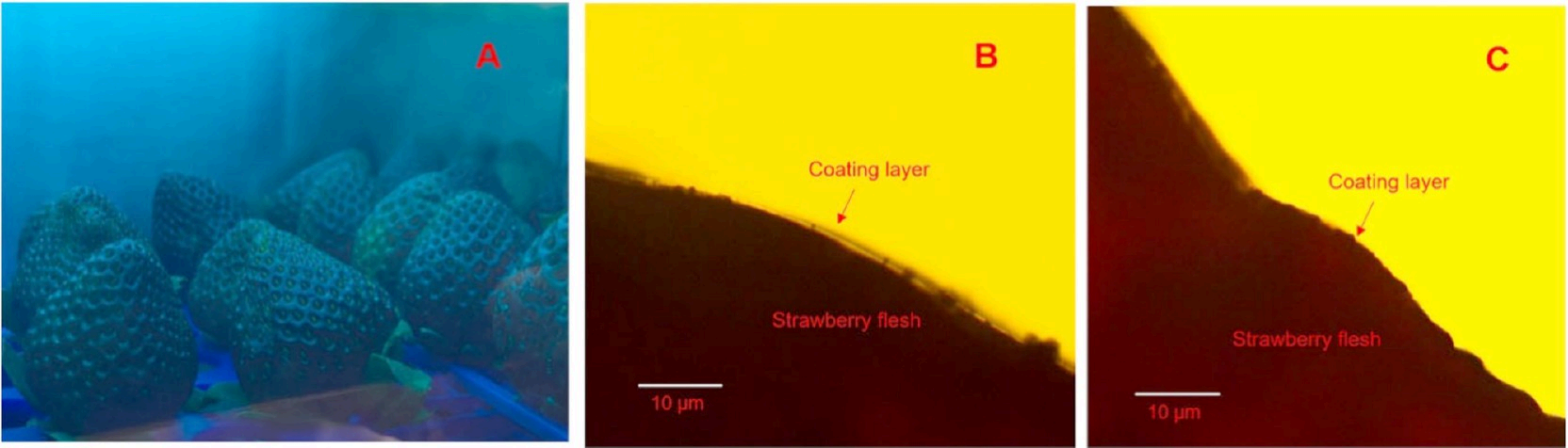
Pictures depicting (a) coated strawberries undergoing UV-A light treatment, (b) a cross section of the coated strawberry slice (magnified 100 times) after 30 min of drying, and (c) a cross section of the coated strawberry slice (magnified 100 times) after 30 min of drying followed by 24 h of refrigerated storage. Adapted with permission from ref (26). Copyright 2022 Elsevier.

Zhang et al. in 2022^26^ developed a novel approach to enhance the safety and extend the shelf life of strawberries by employing photoirradiated chitosan–gallic acid (CS-GA) coatings. Following 180 min of exposure to 360 nm UV-A radiation, these coatings exhibited robust antibacterial activity against the pathogen *E. coli* O157:H7, resulting in a significant reduction of 2.3 ± 0.4 logs in the bacterial count. Transmission spectra from 200 to 800 nm were analyzed using a SpectraMax M5e spectrometer for coatings and coating solutions air-dried for 48 h. UV-A light transmission through the coatings ranged from 58.6 to 82.2%, enabling photochemical reactions. Solidified CS and CS-GA coatings showed higher transmittances at 360 nm compared to their solutions, with minor differences among groups. UV-A treatment slightly reduced the transmittance from 79.7 to 61.0%. The coatings blocked UV-C light (240 nm) while remaining transparent to visible light (600 nm), indicating their potential to protect strawberries without altering their appearance. After coating solutions were applied to fresh strawberries and the strawberries were subjected to UV light treatment, no visible changes to their appearance were observed. Initially, a thin, transparent coating layer was visible under both light and confocal microscopes. However, after UV light exposure, the coating layer thinned, becoming barely detectable under light microscopy, likely due to dehydration. Confocal microscopy showed that the coating material was distributed uniformly on the strawberry surface, although areas such as achenes and convolutions were not fully covered, potentially affecting the antimicrobial effectiveness (Figure 3). GA and CS were dissolved in an aqueous solution of glacial acetic acid during the preparation process, followed by meticulous centrifugation, mixing, and stirring to ensure stability and homogeneity. Coated strawberries demonstrated a significant reduction in bacterial contamination without compromising fruit firmness or mold decay incidence, indicating potential improvements in both safety and quality aspects. Moreover, the research sheds light on the underlying mechanism of action by defining how the combined effects of CS, GA, and ROS generated through UV-A radiation exposure synergistically enhance the antimicrobial effectiveness of the coating. The pH and total soluble solids (TSS) values of all groups were generally stable, with the control group at day 0 showing lower values than coated berries. Titratable acidity in the control group increased from 0.9 to 1.2 by day 14, higher than in the treated groups. This suggests untreated strawberries undergo more chemical changes than light-treated or coated ones. The patterns align with previous studies, indicating that photoirradiated coatings had the least effect on the chemical properties of strawberries. Although the coatings had minimal impact on fruit color and the frequency of mold deterioration, their ability to maintain consistent chemical properties over time during storage indicates their potential value in food safety strategies. This thorough examination emphasizes the importance of photoirradiated CS-GA coatings as a promising intervention for mitigating the risks of foodborne illness associated with strawberries, while also identifying areas for further improvement and optimization in future research endeavors.^26^

**Figure 4:**
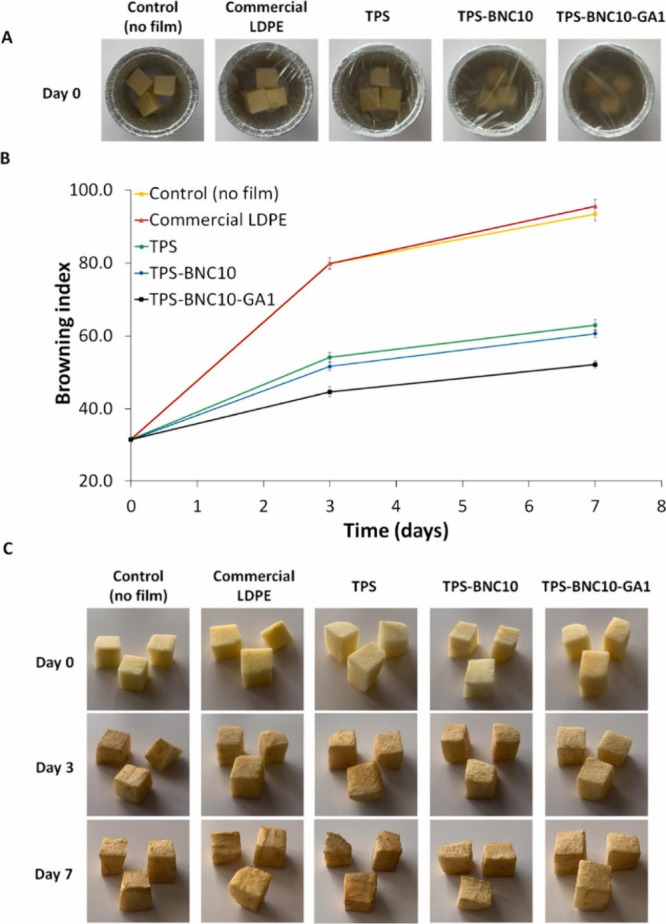
(a) Digital images of the packaged fruits using various films on day 0. (b) Bacterial index (BI) and (c) visual appearance of freshly cut apples before and after storage at +4 °C for 3 and 7 days, either without film or packaged using different films. Adapted with permission from ref (46). Copyright 2023 Elsevier.

Almeida et al. in 2023^46^ synthesized nanocomposite films using thermoplastic starch (TPS) as the base material. These films were reinforced with bacterial nanocellulose (BNC) at concentrations of 1, 5, and 10% by weight relative to starch. Additionally, GA at concentrations of 1 and 1.5% by weight relative to starch was added, and the preparation was carried out through the solvent casting method. The increase in concentration of BNC is attributed to the increase in the tensile strength (TS) and Young’s modulus (YM) of starch film, and this is due to hydrogen bond formation between TPS and BNC.^43^ The modest rise in TS and YM was observed with the addition of GA. TPS, BNC, and GA form strong intermolecular hydrogen bonding resulting in a firm structure.^44^ The moisture absorption ability of TPS has been reduced by the addition of both BNC and GA. Transmittance spectra and opacity values of synthesized films reveal that on increasing the BNC content transparency declined, and the GA content had no effect on these values. On analyzing TGA results, it was clear that BNC and GA content did not affect the decomposition temperature of TPS. UV blocking and antioxidants were exhibited because of the addition of GA. The films without GA did not show any antioxidant activity, but all TPS-BNC-GA films exhibited around 85% antioxidant activity. When the antibacterial activities of TPS and TPS-BNC10 films were evaluated for *Staphylococcus aureus*, they did not decrease the initial bacterial levels, whereas TPS-BNC10-GA1 resulted in substantial initial bacterial concentration reductions of ∼1.6 log CFU mL^–1^ after 24 h and ∼4.5 log CFU mL^–1^ after 48 h. This suggests that the film exhibits a bactericidal effect against *S. aureus*, as it achieved a reduction exceeding 3 log CFU mL^–1^ in the initial inoculum after 48 h. The mechanism that causes inhibition is diffusion of GA through the cell membrane causes hyperacidification of the cytoplasm. The acidification within the cell can modify the cell membrane potential, making it more permeable. This, in turn, induces irreversible alterations that ultimately result in cell death.^45^ The pH of fresh-cut apple samples was measured over 7 days at 4 °C. The initial pH was 4.43, and the pH dropped to 4.07. The TPS-BNC10-GA1 nanocomposite film showed the smallest pH decrease, suggesting its superior performance in preserving apple quality and preventing browning compared to other films (Figure 4). The fresh-cut apple packed in TPS-BNC10-GA1 exhibited lower browning; this was because of the antioxidant ability of GA. The generated TPS-BNC-GA nanocomposite films are promising novel, flexible, eco-friendly, and sustainable active packaging materials.^46^

**Scheme 1:**
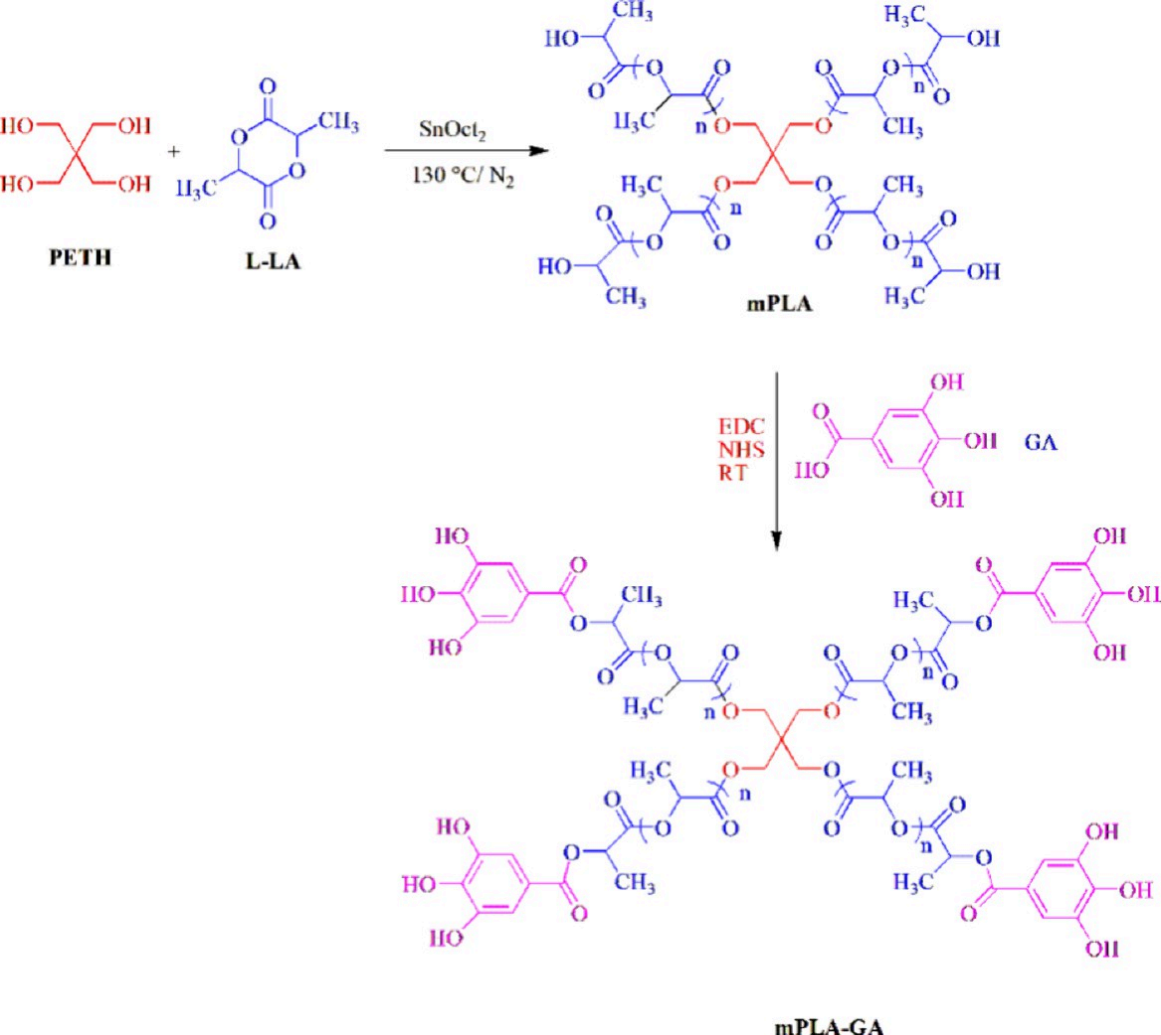
Synthesis Route for mPLA-GA

Taechutrakul and team in 2023^32^ synthesized multibranched PLA functionalized with gallic acid (mPLA-GA) for banana preservation (*Musa* AAA group). The multibranched PLA (mPLA) was created by polymerizing l-lactide (l-LA) using ring-opening polymerization (ROP) on the four-arm pentaerythritol (PETH) core molecule. On incorporation of GA into it, we obtained mPLA functionalized GA (mPLA-GA; Scheme 1). On incorporation of PLA into mPLA-GA using melt processing, PLA/mPLA-GA was made, and the GA content was varied from 1, 2 and 4 wt % during preperation. On analyzing antioxidant activity, it was seen that pure GA demonstrated a quick 90% DPPH scavenging capacity in just 1 min and a 100% capacity after 5 min. The mPLA had no antioxidant activity, and mPLA-GA had a DDPH scavenging ability of 80% in 1 min and 90% in 10 min. This suggests that conjugating GA onto mPLA reduces its migration due to the higher molecular weight of the additive. When the mechanical characteristics were examined, on increasing the GA concentration from 1 to 4%, the strength of PLA/GA decreased from an initial value of 53 ± 4 MPa to approximately 40 MPa. The reason contributing to this behavior is heterogeneous compounding and phase separation between GA and PLA. The tensile strength was improved for PLA/m PLA-GA films by 65 ± 2 MPa for 2% film. On incorporating mPLA/GA, a 20% increase in tensile strength was observed. For studying O_2_ scavenging, PLA/GA 1% was selected, and the test was conducted for 10 days and the remaining concentration of O_2_ in the vial was observed after 10 days. PLA film exhibited less than 2.5%, and PLA/GA exhibited ∼20% oxygen scavenging capacity. PLA/GA exhibited higher oxygen scavenging activity until 30 h, and then it reduced due to a decrease in free GA. PLA/mPLA-GA film showed 13% in the first 20 h and ∼13 to ∼17% in 20–120 and 120–250 h, which showed 17% itself. TGA analysis revealed that mPLA-GA had increased weight loss from 0 to 100 °C due to water absorption by GA’s −OH groups. mPLA decomposed mainly between 253 and 327 °C, with *T*_onset_ at 242 °C, *T*_d10%_ at 253 °C, and *T*_d_ at 306 °C. For mPLA-GA, these values decreased to 244, 233, and 305 °C, respectively, indicating lower thermal stability and confirming GA’s successful integration.

**Figure 5:**
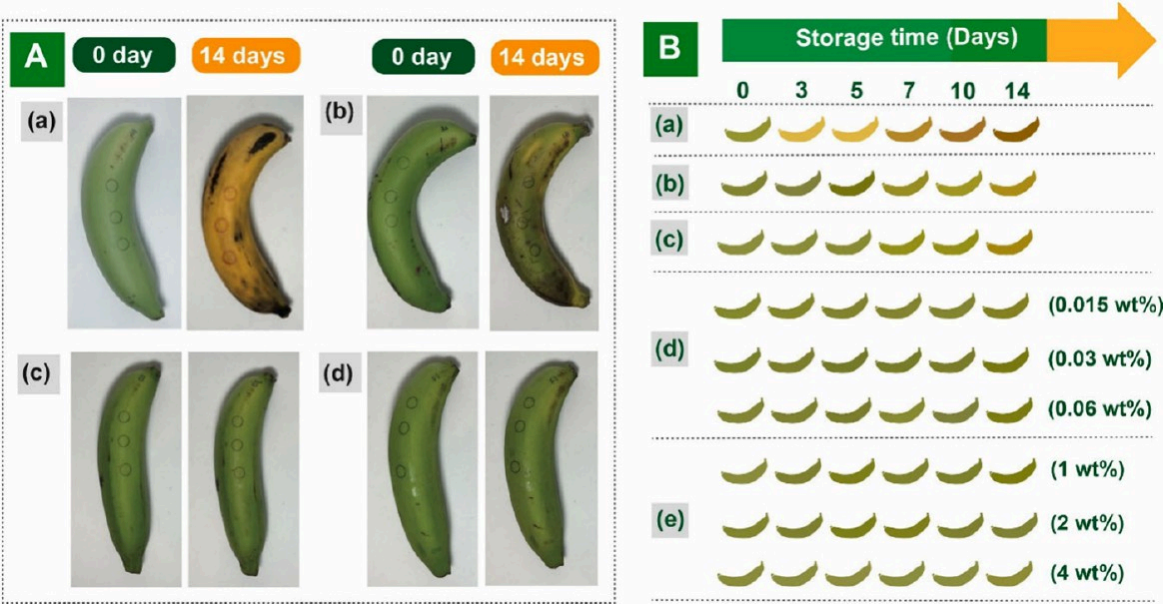
(A) Visual representations of bananas: (a) without packaging in a regular environment, (b) packaged with PLA film strip, (c) packaged with PLA/GA 0.03 wt % film strip, and (d) packaged with PLA/mPLA-GA 2 wt % film strip. (B) Depiction of banana colors: (a) without packaging in a regular environment, (b) packaging without film strip, (c) packaging with PLA film strip, (d) packaging with PLA/GA film strip featuring varying GA contents, and (e) packaging with PLA/mPLA-GA film strip featuring varying mPLA-GA contents. Adapted with permission from ref (32). Copyright 2023 Elsevier.

Bananas were vacuum packed in packaging, and PLA/mPLA-GA film strips were kept for analyzing their potential to extend the shelf life of bananas (Figure 5). The banana kept as a control showed a color change from green to yellow on the 14th day of observation. The banana with PLA film showed a green to patchy yellow color, whereas with the PLA/mPLA-GA strip it exhibited a persistent green color. The findings indicate that PLA/mPLA-GA film strips demonstrated promising oxygen scavenging and antioxidant activity, effectively delaying the ripening process and preventing bananas from browning. The ripening index (RI) indicates the ripening level. The ripening and alteration in the peel color of bananas occur because of chlorophyll breakdown facilitated by the chlorophyllase enzyme. This breakdown leads to increased levels of pigments, including carotene and xanthophyll, which results in a transition from a green to a yellow color. For bananas that were not packed, the RI value was −0.4 on day 0 and −0.1 on day 14. For bananas in PLA/GA and PLA/m PLA-GA films, the RI was very insignificant (∼0.4). The oxygen absorption capacity was analyzed, and it was observed that PLA/mPLA-GA had higher oxygen scavenging ability than the rest. Migration testing of PLA-based films demonstrated that, while mPLA-GA showed no migration into food simulants, free GA migrated significantly into methanol. This difference highlighted the effectiveness of conjugating GA onto mPLA, increasing its molecular weight and preventing migration into food simulants. The study also found that free GA migrated primarily into methanol but not into ethanol, indicating that the higher molecular weight of mPLA-GA effectively hindered migration, ensuring its suitability for food contact applications. The PLA/mPLA-GA film strip demonstrated its advantages and promising potential as a novel active film strip for prolonging the shelf life of bananas. The functional properties of PLA/mPLA-GA make it suitable for food packaging.^32^

## 4 Meat and Fish Products

Edible coating was developed using gallic acid/chitosan (CS/GA) by Fang et al. in 2018 for the preservation of pork.^51^ The modified atmosphere packaging (MAP) technique was used for the synthesis. MAP is employed to partially restrain aerobic and anaerobic microorganisms, prolonging shelf life and also enhancing the fresh appearance of meat. To 1% (w/v) acetic acid solution was added 2% CS, and the solution was stirred overnight. GA (0, 0.2, and 0.4% (w/w)) was incorporated to produce CS, CS/0.2GA, and CS/0.4GA coating solutions. Fresh pork steaks of 5 cm thickness were dipped in the prepared solutions for 1 min, and steaks placed in trays were brought into contact with a 80% O_2_ and 20% CO_2_ mixture. Oxygen-impermeable films were used for hermetic sealing. The pork samples were coated with solutions, and their pHs were analyzed (Table 1). In the control, the decrease in pH is due to the increased growth of lactic acid bacteria (LAB) in pork. At the same time, the presence of CS, which has inherent antibacterial activity, inhibits the growth of bacteria in other samples.^47^ The pH of the pork loin samples was affected by coating treatments and storage duration. Over 20 days, the pH of noncoated samples decreased from 5.51 to 5.43, while those of chitosan-coated samples (CS, CS/0.2GA, CS/0.4GA) increased to pH 5.80, 5.82, and 5.89, respectively. This rise in the pH for coated samples is attributed to protein hydrolysis by endogenous proteases and microbial enzymes. The lower pH in control samples is likely due to higher lactic acid bacteria growth, as chitosan exhibits antimicrobial properties. The main pH changes occurred between days 15 and 20, with no significant difference among chitosan-coated samples, indicating that GA addition did not affect the pH during storage.^48^

**Table 1:** Impact of Gallic Acid/Chitosan Coating on the pH, TBARS, TVC, and Protein Oxidation (Free Thiol Group Values, nmol of Thiol/mg of Protein) of Pork Loin during Modified Atmosphere Packaging (MAP) storage at 4 °C

		days stored				
treatment	sample	0	5	10	15	20
pH	control	5.51 ± 0.06^Aab^	5.53 ± 0.06^Aab^	5.62 ± 0.18^Aa^	5.53 ± 0.04^Cab^	5.43 ± 0.08^Bb^
	CHI	5.51 ± 0.06^Ac^	5.41 ± 0.06^Ac^	5.58 ± 0.08^Abc^	5.70 ± 0.08^Babc^	5.80 ± 0.19^Aa^
	CHI/0.2G	5.51 ± 0.06^Ac^	5.46 ± 0.05^Ac^	5.67 ± 0.09^Abc^	5.76 ± 0.08^Bab^	5.82 ± 0.03^Aa^
	CHI/0.4G	5.51 ± 0.06^Ab^	5.51 ± 0.05^Ab^	5.64 ± 0.11^Aab^	5.89 ± 0.13^Aa^	5.89 ± 0.10^Aa^
TVC (log CFU/g)	control	2.56 ± 0.06^Ae^	4.41 ± 0.06^Ad^	5.28 ± 0.14^Ac^	6.15 ± 0.15^Ab^	6.39 ± 0.06^Aa^
	CHI	2.56 ± 0.06^Ae^	4.03 ± 0.06^Bd^	4.63 ± 0.17^Bc^	4.97 ± 0.13^Bb^	5.16 ± 0.14^Ba^
	CHI/0.2G	2.56 ± 0.06^Ac^	3.96 ± 0.04^Bb^	4.44 ± 0.23^Ba^	4.60 ± 0.25^Ca^	4.60 ± 0.18^Ca^
	CHI/0.4G	2.56 ± 0.06^Ac^	3.95 ± 0.04^Bb^	4.60 ± 0.12^Ba^	4.70 ± 0.16^Ca^	4.61 ± 0.24^Ca^
TBARS	control	0.081 ± 0.06^Ae^	0.199 ± 0.055^Ad^	0.272 ± 0.074^Ac^	0.535 ± 0.012^Ab^	1.004 ± 0.055^Aa^
	CHI	0.081 ± 0.06^Ac^	0.214 ± 0.051^Ab^	0.285 ± 0.060^Ab^	0.451 ± 0.087^Ba^	0.550 ± 0.015^Ba^
	CHI/0.2G	0.081 ± 0.06^Ac^	0.204 ± 0.032^Ab^	0.239 ± 0.006^Aab^	0.250 ± 0.052^Cab^	0.252 ± 0.018^Ca^
	CHI/0.4G	0.081 ± 0.06^Ac^	0.197 ± 0.058^Ab^	0.227 ± 0.015^Aab^	0.242 ± 0.013^Cab^	0.279 ± 0.050^Ca^
protein oxidation (nmol of thiol/mg of protein)	control	69.17 ± 2.38^Aa^	64.80 ± 6.28^Aa^	58.89 ± 1.43^Aa^	47.12 ± 1.43^Cc^	35.69 ± 3.31^Cd^
	CHI	69.17 ± 2.38^Aa^	62.21 ± 1.62^Aa^	57.48 ± 4.12^Aa^	53.68 ± 6.35^ABc^	45.23 ± 4.16^Bd^
	CHI/0.2G	69.17 ± 2.38^Aa^	66.31 ± 1.88^Aa^	57.46 ± 1.31^Aa^	57.95 ± 4.27^Ab^	49.00 ± 0.65^Ac^
	CHI/0.4G	69.17 ± 2.38^Aa^	65.90 ± 2.00^Aa^	55.24 ± 3.80^Aa^	52.13 ± 3.54^Bb^	44.49 ± 1.15^Bc^

On analyzing the color, the CS/GA coating has slowed the increase in lightness (L* value), delayed the decrease in redness (a* value), and maintained lower hue angle values throughout storage. This is due to the antioxidant activity of GA, which retards the oxidation of oxymyoglobin to metmyoglobin. Also, it was observed that CS/0.4GA showed significantly better results than CS/0.2GA. In studying the TVC, the permitted level is less than 7 log CFU/g in pork, and all the samples had a value less than the permissible limit even after 20 days. The chitosan content in the coating has reduced the growth of microorganisms in pork, and the addition of GA has further reduced the TVC in pork. The increased antimicrobial efficacy attained by incorporating GA is likely attributed to its capacity to decrease negative charges, which leads to inducing cell ruptures or pore formation. Consequently, this process leads to the death of microorganisms.^45,47^ The TBARS value was analyzed, and the limit is 0.6 mg of MDA/kg. Incorporation of GA has lowered the TBARS value more than chitosan alone; this is because of the antioxidant ability and increased oxygen barrier ability of GA.^49,50^ The hardness of pork samples was measured by the Warner–Bratzler shear force (WBSF), which states that higher shear force values indicate tougher meat. Initially, the shear force was about 200 N, increasing to 299 (CON), 264 (CS), 253 (CS/0.2GA), and 273 N (CS/0.4GA) by day 20. Protein cross-linking during storage contributed to meat toughening. CS/0.2GA samples showed the lowest shear force on days 15 and 20, suggesting improved tenderness due to delayed lipid and protein oxidation. However, higher gallic acid concentration (CS/0.4GA) resulted in increased shear force, possibly due to prooxidant effects, reducing tenderness. Protein oxidation is a measure of the free thiol group. As cysteine oxidation in meat increases, free thiol groups get reduced, indicating more protein oxidation. The antioxidant activity of the coating was well-established after 15 days, and this may be due to incorporation of the MAP technique. This study proposed that CS/GA coupled with MAP could be used in the future for preserving fresh pork. This approach aims to enhance both the safety and quality of the food product.^51^

In the year 2019, Cao et al. prepared gallic acid/nisin/chitosan coating with the high oxygen modified atmosphere packaging technique (HOMAP) for the preservation of pork.^54^ Using the technique of HOMAP, high oxygen content comes into contact with the meat, due to which protein and lipid oxidation happens and thereby the quality of meat is diminished. Due to the reason herein, they have incorporated antioxidant additives like GA to decrease spoilage. Chitosan (CS), chitosan/nisin solution (CS/N), chitosan/gallic acid solution (CS/GA), and chitosan/nisin/gallic acid solution (CS/N/GA) were prepared. The lightness (L*) of all samples increased over time, indicating that the meat became paler. The CS/N/GA coating slowed this increase compared with other treatments. Redness (a*) peaked at day 5 for all samples due to exposure to high oxygen environments, forming oxymyoglobin. After day 10, redness decreased in CON (noncoated sample), while CS/GA and CS/N coated samples maintained higher redness values throughout the 20 days. This suggests that incorporating GA and N in CS coatings can inhibit pork discoloration under HOMAP. The hue angle increased in CON after 15 days, indicating a shift toward yellow and metmyoglobin formation. Coated samples either prevented or delayed this increase. During 20 days of HOMAP storage, the pH of noncoated pork samples decreased from 5.54 to 5.37 due to LAB growth, while chitosan-coated samples (CS, CS/GA, CS/N, CS/N/GA) showed increased pH values (5.77, 5.79, 5.75, 5.67) attributed to antimicrobial properties of the coatings against spoilage bacteria. No significant pH changes were observed within the first 10 days for all samples.^52^ On studying the TVC, even after 20 days, all samples had values less than the limit of 7 log CFU/g.^53^ Lipid oxidation in pork was examined by the TBARS value. The control showed a TBARS value higher than the limit during storage for 20 days, while pork samples coated with CS, CS/GA, CS/N, and CS/N/GA had a value within the prescribed limit even after 20 days (Table 2). The sample with GA had a lower TBARS than non gallic acid ones, and this may be attributed to the excellent antioxidant activity of GA. GA scavenges the available free radicals and inhibits the oxidation chain reaction.^50^ It was also seen that incorporating GA into CS film could lead to a decrease in oxygen permeability (OP), thereby minimizing pork exposure to oxygen and effectively reducing lipid oxidation. The Warner–Bratzler shear force (WBSF) indicates that the tenderness of pork, which is due to the cross-linking of protein moieties, was declining. The initial shear force value of the pork sample was 182 N. By day 20, this value increased to around 280 N for CON, 255 N for CS, 241 N for CS/GA, 250 N for CS/N, and 236 N for CS/N/GA. The presence of GA slowed the process of hardening. Protein oxidation was examined, and it was seen that, since the HOMAP technique was incorporated, the high oxygen atmosphere induced intermolecular cross-linking of protein molecules and protein oxidation was reduced for 20 days. Here in this study, the use of CS/GA and N in the manufacture of coating with the incorporation of the HOMAP technique paves the way for effective meat conservation.^54^

**Table 2:** Impact of GA/N/CS Coating and Duration of Storage on the pH, TVC, TBARS, and Protein Oxidation (Free Thiol Group Values) of Pork Loins under HOMAP (80% O_2_; 20% CO_2_) Stored at 2 ± 1 °C

		days stored				
treatment	sample	0	5	10	15	20
pH	control	5.54 ± 0.05^Aa^	5.53 ± 0.02^Aa^	5.49 ± 0.02^Bab^	5.45 ± 0.04^Cab^	5.37 ± 0.05^Cb^
	CS	5.54 ± 0.05^Abc^	5.58 ± 0.01^Ac^	5.58 ± 0.03^Abc^	5.65 ± 0.01^BCb^	5.77 ± 0.05^Aba^
	CS/GA	5.54 ± 0.05^Ac^	5.57 ± 0.03^Ac^	5.66 ± 0.04^Abc^	5.74 ± 0.02^Aab^	5.79 ± 0.03^Aa^
	CS/N	5.54 ± 0.05^Ac^	5.58 ± 0.02^Ac^	5.64 ± 0.02^Abc^	5.68 ± 0.01^Bab^	5.75 ± 0.04^Aba^
	CS/N/GA	5.54 ± 0.05^Ab^	5.53 ± 0.03^Ab^	5.64 ± 0.02^Abc^	5.65 ± 0.04^ABa^	5.67 ± 0.02^Ba^
TVC (log CFU/g)	control	2.71 ± 0.02^Ae^	4.41 ± 0.01^Ad^	5.55 ± 0.01^Ac^	6.31 ± 0.02^Ab^	6.83 ± 0.03^Aa^
	CS	2.71 ± 0.02^Ae^	3.56 ± 0.12^Cd^	4.58 ± 0.02^Bc^	5.53 ± 0.12^BCb^	6.41 ± 0.07^Ba^
	CS/GA	2.71 ± 0.02^Ae^	3.63 ± 0.16^Cd^	4.37 ± 0.03^Cc^	5.49 ± 0.01^Bb^	5.76 ± 0.07^BCa^
	CS/N	2.71 ± 0.02^Ae^	4.14 ± 0.02^Bd^	4.54 ± 0.02^Bc^	5.32 ± 0.03^Cb^	5.95 ± 0.15^Ca^
	CS/N/GA	2.71 ± 0.02^Ae^	3.48 ± 0.13^Cd^	3.99 ± 0.02^Dc^	4.31 ± 0.05^Db^	4.67 ± 0.07^Da^
TBARS (mg of MDA/kg)	control	0.07 ± 0.01^Ae^	0.22 ± 0.02^Ad^	0.33 ± 0.01^Ac^	0.50 ± 0.03^Ab^	1.08 ± 0.03^Aa^
	CS	0.07 ± 0.01^Ae^	0.16 ± 0.02^Ab^	0.18 ± 0.01^Cb^	0.28 ± 0.03^Ba^	0.33 ± 0.01^Ba^
	CS/GA	0.07 ± 0.01^Ae^	0.08 ± 0.01^Bc^	0.10 ± 0.03^Dbc^	0.16 ± 0.02^Cb^	0.28 ± 0.01^Ca^
	CS/N	0.07 ± 0.01^Ae^	0.22 ± 0.02^Ac^	0.25 ± 0.02^Bbc^	0.31 ± 0.02^Bab^	0.33 ± 0.01^Ba^
	CS/N/GA	0.07 ± 0.01^Ae^	0.09 ± 0.01^Bb^	0.10 ± 0.02^Db^	0.26 ± 0.02^Ba^	0.28 ± 0.02^Ca^
protein oxidation (nmol of thiol/mg of protein)	control	67.04 ± 2.95^Aa^	59.88 ± 1.86^Ab^	52.32 ± 1.12^Bc^	40.33 ± 2.77^Bd^	33.09 ± 2.24^De^
	CS	67.04 ± 2.95^Aa^	58.49 ± 1.68^Ab^	53.01 ± 1.29^ABc^	46.89 ± 3.52^ABcd^	43.18 ± 1.09^BCd^
	CS/GA	67.04 ± 2.95^Aa^	62.36 ± 0.84^Aa^	56.63 ± 1.27^Ab^	49.51 ± 1.04^Ac^	46.25 ± 0.45^Ad^
	CS/N	67.04 ± 2.95^Aa^	61.11 ± 0.98^Ab^	54.95 ± 1.03^ABc^	47.54 ± 0.53^Ad^	44.57 ± 1.12^ABe^
	CS/N/GA	67.04 ± 2.95^Aa^	62.86 ± 1.42^Aa^	53.89 ± 0.49^ABb^	49.64 ± 0.89^Ac^	47.07 ± 0.35^Ad^

**Figure 6:**
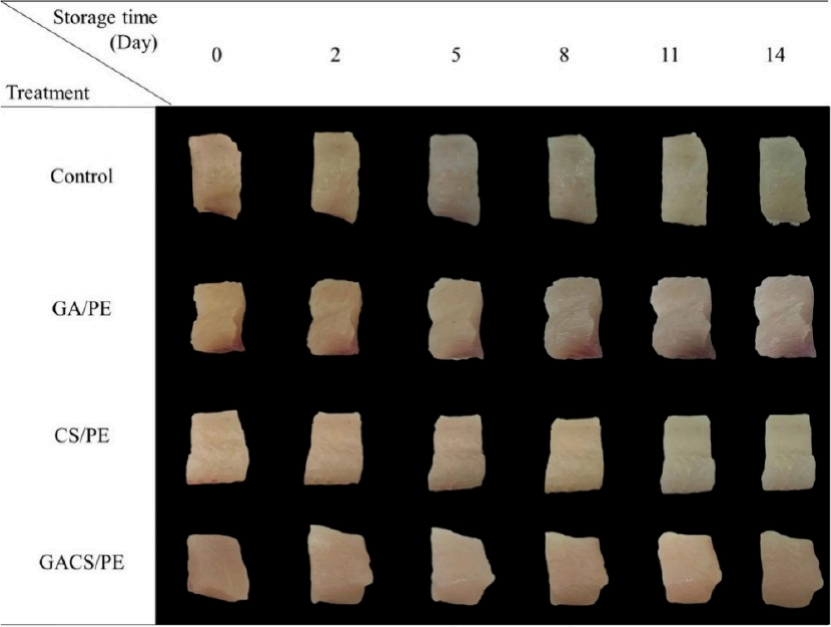
Visual assessment of tilapia stored under various packaging conditions at 4 °C for 14 days. Adapted with permission from ref (33). Copyright 2020 Elsevier.

Tilapia (*Oreochromis niloticus*) fillet preservation at 4 °C by polyethylene (PE) film coated by chitosan/gallic acid was studied by Wong et al. in 2020.^33^ The plasma treatment method was adopted for coating CS/GA in PE films. Four films were synthesized: control (PE films), plasma-treated PE coated with GA (GA/PE), plasma-treated PE coated with CS (CS/PE), and plasma-treated PE coated with GA and CS (GACS/PE). GACS/PE films had the highest antioxidant activity compared to the rest. Control and CS/PE had similar radical scavenging activities. The GA/PE films could scavenge 37%, whereas GACS/PE could scavenge 90%, which indicates the excellent antioxidant capacity of GACS. The TPC was calculated. On analyzing on day 11, it was seen that both the control group and the GA/PE group exceeded the specified limit, recording bacterial counts of 6.02 and 5.43 log CFU/g of meat, respectively. In contrast, the CS/PE and GACS/PE groups experienced a delay of 3 days before reaching this limit, ultimately attaining bacterial counts of 5.05 and 4.97 log CFU/g of meat, respectively. It was observed that the antimicrobial abilities of GACS/PE and CS/PE were similar, indicating that GA made no significant contribution to the antimicrobial activity. TVBN formation in fish indicates microbial activity in stored fish, forming compounds like ammonia, dimethylamine, and trimethylamine. The allowed limit of TVBN in edible meat is 30–35 mg/100 g.^55^ On day 1 the TVBN count was 1.867 mg/100 g. Control, CS/PE, GA/PE, and GACS/PE exhibited TVBN values of 81.67, 12.93, 56.57, and 8.21 mg/100 g, respectively, on the 14th day. The combined effect of GA and CS was the reason for the lower TVBN value of the GACS/PE film. The formation of thiobarbituric acid (TBA) in fish is also a reason for foul odor. TBA which indicates the level of lipid oxidation in fish is measured, which indicates the level of lipid oxidation in fish. Fresh fish exhibited a TBA value of 0.15 mg of MDA/kg. After 14 days it was observed that control, GA/PE, CS/PE, and GACS/PE showed values of 0.3075, 0.2630, 0.3013, and 0.2307 mg of MDA/kg, respectively. The appearance of fillets was also analyzed, and it was seen that fillets packed in GACS/PE could maintain the color of fillets more effectively than others (Figure 6). The excellent antioxidant ability of the film can be utilized for future use. Also, the incorporation of plasma technology in food technology is a better option.^33^

In 2021 Song et al.^31^ synthesized collagen (COL)/zein (ZN)/gallic acid (GA) films using an electrospinning technique. GA (0, 1, 2, 4, 6, 8, and 10% (w/w)) was incorporated in film synthesis. The DPPH radical scavenging activity indicated the antioxidant activity of the films. COL/ZN (GA0) exhibited 3.75 ± 0.71%, whereas with increasing GA concentration the antioxidant activity was enhanced from 15.25 ± 1.25 to 92.39 ± 0.07%. The antioxidant activities of films with GA8 and GA10 had similar values, and this may be due to the saturation of the available hydroxyl group on the surface of films. Tilapia muscle was packed in the films to analyze its food-preserving capacity. The pH of the tilapia muscle was monitored during storage at 4 °C. Initially, the pH was 6.945. The pH of muscles packaged with GA0 and GA8 films decreased slightly on day 1 due to glycolysis and then increased. The control group’s pH consistently rose. By the end of storage, the GA8 film group had a significantly lower pH compared to control and GA0, indicating GA8 film’s effectiveness in inhibiting enzyme activity and microbial growth due to its antibacterial properties.^56^ The hardness of tilapia was examined, and GA8 prolonged the hardness more compared to the others. GA could effectively prevent muscle protein degradation by inhibiting the breakage of disulfide bonds, thereby reducing the exposure of hydrophilic and hydrophobic groups.^57^ The TVBN was analyzed (limit, 20 mg/100 g). When the TVBN value was measured on the fifth day, control and GA0 exhibited 20.01 and 21.07 mg/100 g, whereas tilapia muscle packed in GA8 film exhibited 21.76 mg/100 g only on the 10th day. This reveals that GA8 film has the potential to hinder protein degradation, possibly due to the breakdown of bacterial cell walls or enzyme systems by GA. When measuring the TVC value, which is a measure of the degree of spoilage, it was clear that GA8 films had an excellent ability to maintain the quality of the fish. For fresh fish, the TVC was 1.69 log CFU/g. On the fifth day, the control and GA0 groups had values of 6.12 and 6.06 log CFU/g, whereas the GA8 group reached the limit of 6 log CFU/g on the 10th day. The presence of GA having antibacterial properties is the reason the TVC content did not increase beyond the limit even on the 10th day. All the studies conducted imply that COL/ZN/GA8 is the better film to increase the shelf life of tilapia fillets.^31^

**Figure 7:**
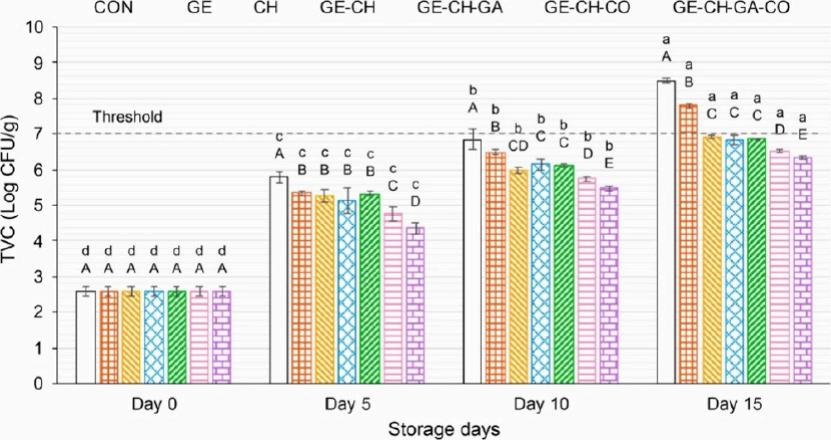
Variations in the total viable count (TVC) of salmon fillet samples during refrigerated storage (4 °C) over 15 days. Adapted with permission from ref (17). Copyright 2021 Elsevier.

In 2021 Xiong et al.^17^ prepared salmon bone gelatin/chitosan/gallic acid/clove oil (GE-CS-GA-CO) edible coating for the preservation of salmon fillets during cold storage (at 4 °C). Gelatin was extracted from salmon bone. The prepared coatings were coated on the fish samples. The initial pH of fresh salmon fillet was 6.22, within the normal range of pH 6.1–6.3. During storage, the pH increased due to nitrogenous compounds from autolysis and microbial activity. Coated samples effectively slowed this increase, staying within the normal pH range by day 5, while the uncoated sample’s pH exceeded it. Chitosan-based coatings (CS, GE-CS, GE-CS-GA, GE-CS-CO, GE-CS-GA-CO) were more effective than gelatin alone, likely due to chitosan’s antimicrobial properties. Gallic acid incorporation further suppressed the pH increase, with the GE-CS-GA-CO coating being the most effective due to synergistic antimicrobial and antioxidant effects. On analyzing the meat color after coating, coated samples showed higher lightness (L*) values compared to the uncoated control. During storage, all samples experienced a decrease in lightness, but coated samples maintained higher L* values throughout, with GE-CS-GA and GE-CS-GA-CO performing best. Redness (a*) decreased in all samples over time, indicating pigment oxidation, but coated samples showed better color retention. Yellowness (b*) increased in all samples, potentially due to lipid oxidation, but coated samples had lower b* values by the end of the storage period. It was observed that the GE-CS-GA-CO coating effectively preserved the color qualities of salmon fillets during cold storage, likely due to the antioxidant and antibacterial properties of GA and CO. TBARS assay was conducted (limit, 1–2 mg of MDA/kg of fish). Initially, the TBARS value was 0.29 mg of MDA/kg in fresh salmon. TBARS levels in all fillet samples increased daily but stayed below 1 mg of MDA/kg for the initial 10 days of storage. For the control, TBARS exceeded the limit after the 10th day, indicating increased lipid oxidation from direct oxygen exposure and free radical production during the lipid chain reaction.^58^ The GE-coated sample exceeded the limit on the 15th day but had a lower TBARS value than the control. The chitosan coating exhibited lower TBARS than gelatin due to chitosan’s excellent antioxidant and gas barrier properties.^59,60^ Also, the addition of GA could boost the coating’s antioxidant properties, as the samples with GA incorporated into the coating exhibited the lowest TBARS values across all samples on days 5, 10, and 15. The incorporation of CO had no significant effect on the lipid oxidation. Protein oxidation was also analyzed by the availability of a free thiol group. On day 0 the free thiol group was 53.85 nmol of thiol/mg. All coatings successfully shielded the salmon fillet from protein oxidation, likely due to their gas and oxygen barrier properties, which prevented the fillet from being directly exposed to oxygen.^61^ Interestingly it was observed that, due to the presence of GA, GE-CS-GA and GE-CS-GA-CO had lower protein oxidation. The TVC content in meat had 7 log CFU/g as the maximum limit.^62^ On initial observation, TVC was found to be 2.60 log CFU/g. Until the 10th day, the TVC count in control was within the acceptable limit, but by the 15th day, it was 8.50 log CFU/g, indicating increased microbial growth in the fillet (Figure 7). The fillet with GE coating also had an increase in TVC content, and it exhibited 7.79 log CFU/g TVC on the 15th day. It was seen that the GE-CS-GA-CO coating exhibited the lowest TVC value among all samples. The notable antimicrobial effect of CO was the reason for this observation. The CO-infused coating, made with GE and/or CS, has been proven to suppress a variety of fish-borne spoilage bacteria such as *E. coli, Bacillus cereus*, *S. aureus*, and *Salmonella* species.^63^ It is proposed that eugenol, the key component of clove oil, might be the primary active compound driving its antimicrobial effects.^64^ The developed coating showed excellent results in the preservation of salmon. GE-CS-GA-CO was the best in preserving the qualities of salmon even for 5 days.^17^

In 2021, Huang et al.^66^ synthesized pectin grafted with gallic acid and propyl gallate for the preservation of fresh bass (*Lateolabrax maculatus*). The synthesized samples were named GA-Pe (pectin modified with gallic acid) and Pr-Pe (pectin modified with propyl gallate). Unmodified pectin (Na-Pe) was also used for comparing the results. On analyzing antioxidant activity, the modified pectin exhibited significant DPPH radical scavenging activity. When compared to Na-Pe, GA-Pe and Pr-Pe showed notably higher clearance rates of 70.9 and 69.7%, respectively. In this the DPPH radical was neutralized by the donation of hydrogen atoms, resulting in the formation of a stable DPPH–H molecule.^65^ Na-Pe also showed a decrease in β-carotene bleaching, but GA-Pe and Pr-Pe showed 45.54 and 78.99%, respectively. The electron or hydrogen donation capability of phenolic hydroxyl groups introduced by GA or Pr played a crucial role in the antioxidant activity of modified pectin. On analyzing antibacterial activity, small inhibition zones were observed for Na-Pe and GA-Pe, whereas Pr-Pe showed excellent antibacterial activity. Also, the inhibition zone shown by Pr-Pe against *E. coli* was better compared to *S. aureus*, and this may be attributed to the lighter peptidoglycan layer in Gram-negative bacteria (*E. coli*) compared with Gram-positive bacteria (*S. aureus*). Na-Pe, GA-Pe, and Pr-Pe were coated on bass fillet. It was refrigerated, and its properties were analyzed. The TVC was measured, and it was seen that Na-Pe and control had very similar TVC values and Pr-Pe exhibited the lowest TVC value. Histamine content in fish reveals the amount of fish spoilage. Control and Na-Pe had higher histamine contents during the storage process. Pr-Pe had a significant impact in reducing histamine levels compared to GA-Pe. Lipid peroxidation is usually measured by analyzing the MDA content in fish, and for this purpose the TBA value is noted. The TBA value showed the most pronounced change in the control and Na-Pe groups, whereas the GA-Pe and Pr-Pe groups effectively prevented the rise in MDA levels. This may be attributed to the excellent antioxidant characters of GA and Pr. Interestingly, the TBA value of Pr-Pe on day 10 was almost similar to the ones on day 0 and day 5, showing the excellent antioxidant activity of Pr. The acid value (AC) was measured, which implies the degree of fat oxidation. On days 5 and 10, the AC of the Na-Pe group was slightly higher than that of the control group. However, the GA-Pe and Pr-Pe groups significantly lowered the AC in bass fillet samples compared with the control. Sample coated with Pr-Pe had the lowest AC value, indicating low fat oxidation in fillet samples. Though GA-Pe and Pr-Pe exhibited good preservation capacity, it was noted that the presence of Pr imparted better properties than GA to pectin.^66^

Gallic acid (GA) induced Chinese yam starch (YS)/chitosan (CS) films were synthesized for pork preservation by Rong et al. in 2022.^69^ YS was extracted from Chinese yam and then GA-induced YS was synthesized.^67^ A solution of 6% (w/v) GA-induced YS was dispersed in 50 mL of distilled water, and CS of different concentrations (0.1, 0.2, 0.6, and 1% (w/v)) dissolved in 0.1% acetic acid was added. Glycerol (1.5%, w/v) was added as an additive to induce film-forming properties to the solution. YS/CS1, YS/CS2, YS/CS3, YS/CS4, GA/YS/CS1, GA/YS/CS2, GA/YS/CS3 and GA/YS/CS4 films were prepared where CS1, CS2, CS3, CS4 refer to 0.1, 0.2, 0.6, and 1% (w/v) chitosan, respectively. The TS of each film was analyzed, and it was observed that on increasing the CS concentration the TS was increased for YS/CS films. This happens because under subcritical conditions it is possible that the amino group of CS becomes protonated into NH_3_^+^, and this facilitates interaction between CS and starch molecules. Specifically, the NH_3_^+^ of CS establishes hydrogen bonds with the hydroxyl group of starch during the gelatinization process.^68^ But YS/CS3 exhibits greater TS than YS/CS4, and this may be due to the aggregation of CS in films. A similar trend was exhibited by the GA/YS/CS films. The TS of the GA/YS/CS film with a high concentration of CS decreased upon GA addition, which implies that GA treatment may have influenced the ionization of OH groups, thereby inhibiting the interaction between CS and starch. On the addition of CS, the viscosity of the solution prepared for film formation was decreased, and thereby the thicknesses of films were reduced. This remarkably increased the light transmittance of the films.

**Figure 8:**
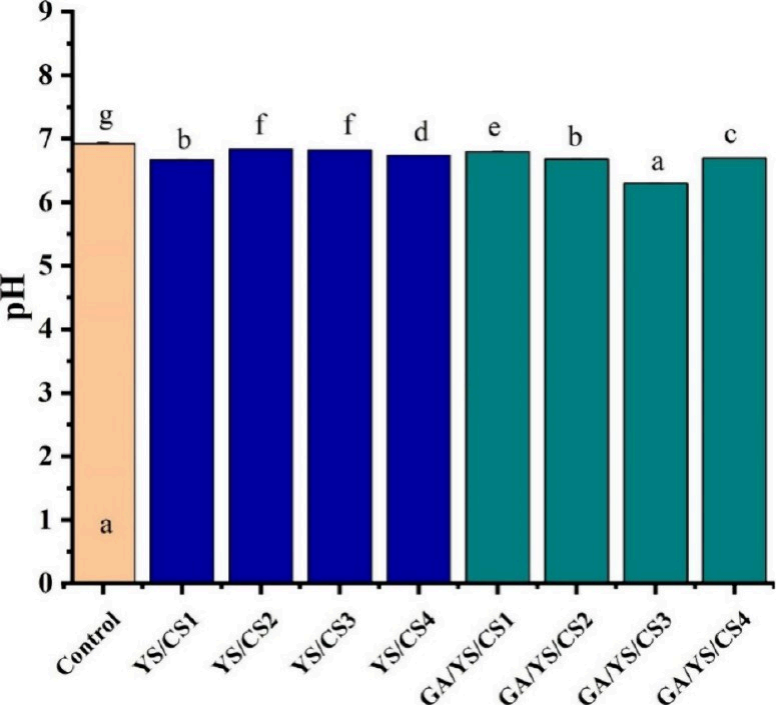
pH values of packaged pork meat after being stored at room temperature for 24 h. Adapted with permission from ref (69). Copyright 2022 Elsevier.

A pork piece packed in polyethylene was taken as the control. The pH values of fresh pork meat were used to evaluate the freshness. Pork packaged in PE film had pH >6.5, indicating spoilage after 24 h at room temperature. In contrast, pork packaged with YS/CS and GA/YS/CS films maintained a lower pH, with GA/YS/CS3 keeping the pH below 6.5 due to its stronger antibacterial properties, enhanced by gallic acid (GA). GA improved the antioxidant activity and solubility of chitosan (CS), contributing to the freshness preservation of the pork (Figure 8). The GA/YS/CS films synthesized exhibited promising applications in pork preservation due to their mechanical and antibacterial properties.^69^

**Figure 9:**
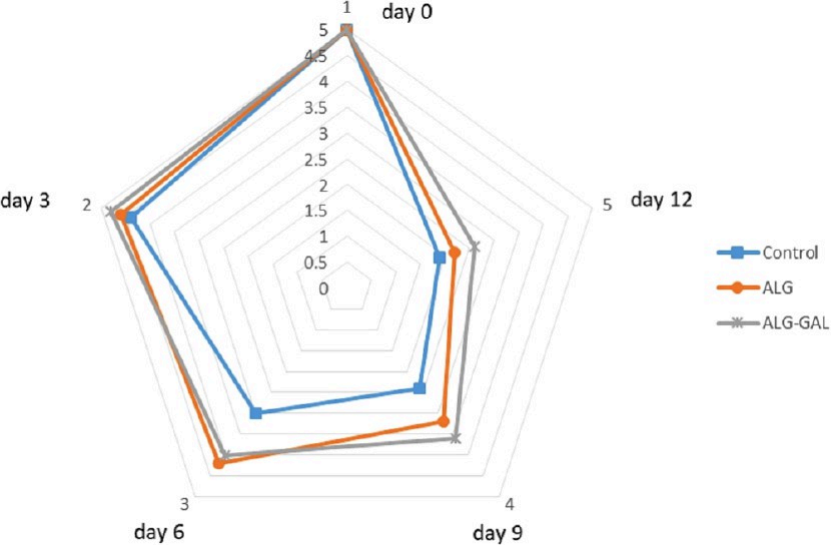
Changes in the sensory evaluation of mackerel fillets during refrigerated storage. Adapted with permission from ref (73). Copyright 2023 Elsevier.

Khodanazary and co-workers in 2023 studied the effect of sodium alginate–gallic acid coating on mackerel fillets (*Scomberomorus commerson*).^73^ The study was conducted for 12 days at 4 °C (Figure 9). Sodium alginate (ALG), and sodium alginate/gallic acid (ALG-GA) were prepared, and fillets were immersed in these solutions for a time period of 30 s. Control, ALG, and ALG-GA were considered for further study. The freshness of seafood, especially of the Scombroidae and Scomberesocidae families, can be assessed through the detection of biogenic amines (BAs), which emit undesirable odors^70^ (limit, 17.96 mg/kg). Histamine (HIS), tyramine (TYR), putrescine (PUT), cadaverine (CD), tryptamine (TRY), 2-phenylethylamine (2-PHE), agmatine, spermine (SM), and spermidine (SD) were the major BAs in this study. On analyzing the histamine content in fish, it was observed that ALG-GA had lower histamine content than the limit even on the 12th day (limit, 50 mg/kg), whereas in the control the histamine content exceeded the limit on the sixth day. The reduced histamine content in mackerel coated with ALG-GA is due to the antibacterial capacity of GA and the oxygen barrier properties of ALG. TRY content was not found in fish until the 12th day. PUT and CD along with HIS are responsible for the pungent odor in fish. Fish coated with ALG-GA had lower PUT, CD, SM, and SD levels than other samples. The biogenic amine index (BAI) was calculated, and samples with ALG-GA coating values did not cross the limit of 20 mg/kg even after 12 days. The TVC was determined and the control, ALG, and ALG-GAL exhibited 3.21, 3.07, and 3.10 log CFU/g TVC values, respectively, on the first day. The control could only be used until day 6 according to the TVC count. By the ninth day, all the treated and nontreated samples had crossed the maximum allowed TVC (limit, 7 log CFU/g). The TVCs of ALG and ALG-GA samples did not exhibit much difference, and their values lower than that of the control are due to the oxygen barrier properties of ALG. Thiobarbituric acid (TBA) is a crucial indicator for assessing the presence of malondialdehyde (MDA), a byproduct of the peroxidation process of unsaturated fatty acids in seafood, and was also analyzed (limit, 1–2 mg of malonaldehyde/kg). The TBA of the control showed an increase day by day. In the addition of GA to ALG, the MDA content was reduced, and this may be attributed to the antioxidant activity of GA.^71,72^ ALG-GA exhibited a lower TBA value than the rest. The developed coating can be used further to prolong the shelf life and prevent the development of any pungent smell throughout the entire storage period.^73^

**Figure 10:**
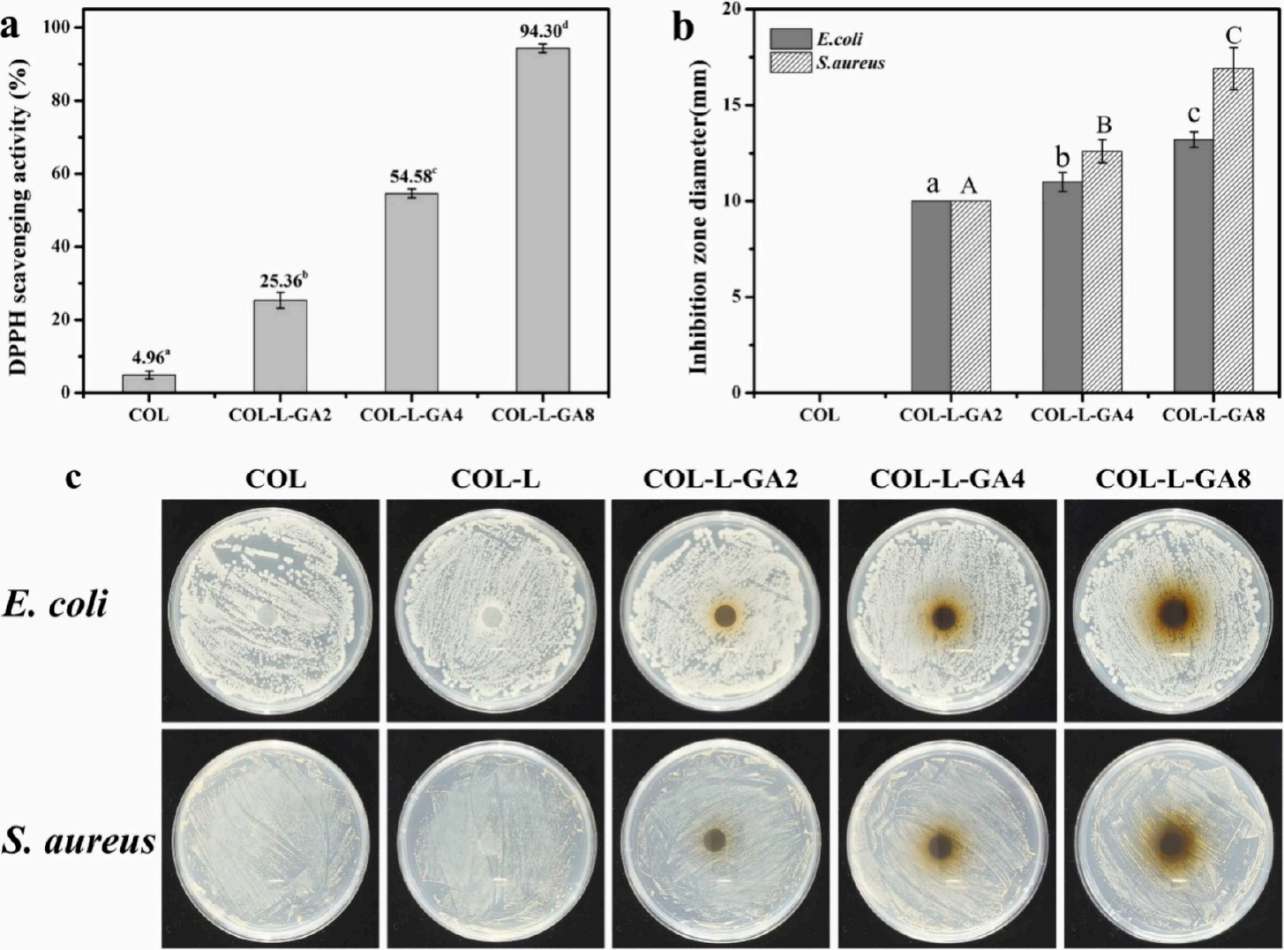
DPPH radical scavenging activity (a), inhibition zone diameter (b), and antimicrobial effectiveness against *E. coli* and *S. aureus* (c) of COL and COL/L/GA films. Adapted with permission from ref (76). Copyright 2022 Elsevier.

Collagen/laccase/gallic acid (COL/L/GA) films were synthesized by Tang et al. in 2023 for beef preservation.^82^ A collagen sponge dissolved in acetic acid of pH 4 was prepared, and glycerol was added as a plasticizer. Laccase (0.2 U/mL) was added, and 2, 4, and 8 mmol of GA were incorporated to produce COL/L/GA2, COL/L/GA4, and COL/L/GA8 films, respectively. The moisture content in COL film was reduced with the addition of GA. The reason for the decrease in hydrophilicity is the formation of cross-links between the NH_2_ of collagen and quinone radicals. Similarly, the water solubility value was reduced with the increasing concentration of GA. The water vapor permeability (WVP) value of COL films was 2.18 × 10^–12^ g·m^–1^·s^–1^·Pa^–1^, and that of film with 8 mmol of GA was 1.73 × 10^–12^ g·m^–1^·s^–1^·Pa^–1^ indicating the reduction in WVP which is good for food packaging applications. The DPPH free radical scavenging rate experienced a notable increase, from 25.36 to 94.30% on increasing the concentration of GA from 2 to 8 mmol (Figure 10). Due to its excellent scavenging ability, COL/L/GA8 can be used for food packaging. The light transmittance of COL/L/GA films was significantly lower than that of COL film, particularly in the UV range, indicating that COL/L/GA films had excellent UV resistance. This enhanced UV blocking capacity of COL/L/GA films was due to the presence of GA, whose aromatic groups could absorb UV light.^74^

On undergoing the DSC experiment, it was observed that with increasing GA concentration the thermostability of films was increasing (Table 3). The denaturation temperature (*T*_d_) points out the collagen denaturation was increasing. In TGA, COL/L/GA8 exhibited the highest heat resistance by a residual mass of 31.02% whereas COL had only 20.67%. This clearly explains the role of GA in increasing the thermal stability of COL films. Similarly, with the addition of GA the tensile strengths of films increased, which may be attributed to the formation of covalent cross-linking between COL and quinones. But, elongation at break showed a decline with increasing GA concentration, and this may be because due to strong interactions molecular chain stretching has been inhibited. On analyzing antibacterial activity, COL films exhibited no antibacterial activity, and on increasing the GA concentration, the inhibitory zone had diameters of 10–13 mm against *S. aureus* and 10–17 mm against *E. coli*. Phenolic compounds have the potential to modify the permeability of bacterial cell membranes and also hinder cell function, ultimately resulting in bacterial death.^75^ The pH of fresh beef was found to be 5.55 on the first day, whereas the beef wrapped in COL/L/GA8 film exhibited pH 6.53 on the 12th day and the pH of control was greater than 7. On analyzing the TVC (limit, 6.00 log CFU/g) on the sixth day, the value of COL/L/GA8 was 5.32 log CFU/g whereas it exceeded the limit for the control and COL groups. The TVBN content was studied. Fresh meat had a value of 7.35 mg/100 g (limit, 15 mg/100 g). On the third day, COL/L/GA8 exhibited 13.07 mg/100 g whereas for beef in the control and COL groups the value exceeded the limit. On the 12th day, beef wrapped in control and COL showed 29.47 and 30.15 mg/100 g. The pH of minced beef was monitored during storage, starting from 5.55. As storage time increased, pH values rose due to nitrogenous substances from protein degradation. The pH of beef wrapped in COL/L/GA8 film increased more slowly, reaching 6.53 on the 12th day, while beef in the COL and control groups exceeded pH 7. The TVBN content in COL/L/GA8 showed only a small increase. Similarly, on analyzing the TBARS value, it was clear that on the 12th day control, COL, and COL/L/GA8 exhibited 0.95, 0.96, and 0.68 mg of MDA/kg. These results prove the importance of COL/L/GA films to be used for food preservation.^76^

**Table 3:** Moisture Content (MC), Water Solubility (WS), Water Vapor Permeability (WVP), Tensile Strength (TS), Elongation at Break (EB), Denaturation Temperature (*T*_d_), and Residual Mass (*M*_r_) values of COL and COL/L/GA Films^a^

treatment	COL	COL/L/GA2	COL/L/GA4	COL/L/GA8
MC (%)	17.11 ± 0.52^d^	16.42 ± 0.40^c^	15.41 ± 0.13^b^	14.21 ± 0.21^a^
WS (%)	22.41 ± 1.30^d^	19.40 ± 0.55^c^	17.76 ± 0.64^b^	15.15 ± 0.91^a^
WVP (×10^–12^ g·m^–1^·s^–1^·Pa^–1^)	2.18 ± 0.03^d^	2.02 ± 0.05^c^	1.88 ± 0.04^b^	1.73 ± 0.06^a^
TS (MPa)	67.19 ± 1.27^a^	78.56 ± 1.83^c^	88.44 ± 1.83^d^	110.20 ± 3.55^e^
EB (%)	40.04 ± 0.98^e^	37.57 ± 0.65^c^	35.88 ± 0.55^b^	33.51 ± 0.74^a^
*T*_d_ (°C)	63.2	69.7	73.7	81.1
*M*_r_ (%)	20.67	24.81	28.05	31.02

a Different letters within the same row indicate significant differences (*p* < 0.05).

Fu et al. in 2023^78^ synthesized gallic acid–modified agarose coating (Ga-Ag) for the preservation of grass carp (*Ctenopharyngodon idellus*). The carbodiimide coupling technique was followed during the synthesis technique. Ga-Ag was synthesized by the EDC·HCl/DMAP method.^77^ Gallic acid (0.3, 0.6, 1.0, and 1.42 g) was incorporated to produce Ga-Ag@3.50%, Ga-Ag@6.50%, Ga-Ag@10.69%, and Ga-Ag@13.73%, respectively. The gelling and melting temperature of the agarose were reduced on incorporation of GA, thereby facilitating the coating application. DPPH scavenging activity and β-carotene bleaching were done to evaluate the antioxidant activity. The native agarose (Na-Ag) showed no scavenging ability on DPPH, whereas GA–modified Ag exhibited scavenging capacities of 16.34% for Ga-Ag@3.50%, 28.63% for Ga-Ag@6.50%, 53.16% for Ga-Ag@10.69%, and 65.92% for Ga-Ag@13.73%, respectively. With increasing grafting percentage, the DPPH scavenging ability also increases. Antioxidant assays not only validated the modified agarose’s radical scavenging ability but also demonstrated its capacity to adsorb onto the surface of oleic acid, inhibiting its oxidation and thereby safeguarding β-carotene from bleaching. The plate coating method was carried out to analyze the antibacterial activity. On carrying out incubation for 24 h, modified Ag as well as GA/Ag blends all demonstrated a 100% inhibition ratio against *E. coli*, compared to native agarose. However, after incubation for 3 days, the inhibition ratios of modified Ag and GA/Ag blends declined from 100% to 38.6% for Ga-Ag@3.50%, 57.9% for Ga-Ag@6.50%, 16.7% for Ga/Ag@3.50%, and 33.3% for Ga/Ag@6.50%, respectively. However, the inhibition ratios of gallic acid/agarose blends (Ga-Ag@3.50%, Ga-Ag@6.50%) decreased from 32.8 and 98.5% to 23.1 and 90%, respectively, against *S. aureus*. The antibacterial activity of the agarose was improved by the incorporation of GA. Compared to GA/Ag blends, GA–modified Ag showed a greater inhibition ratio and a longer inhibitory period. The bacteriostatic action of the gallic acid/agarose blends may have been impacted by their lack of homogeneity.

**Figure 11:**
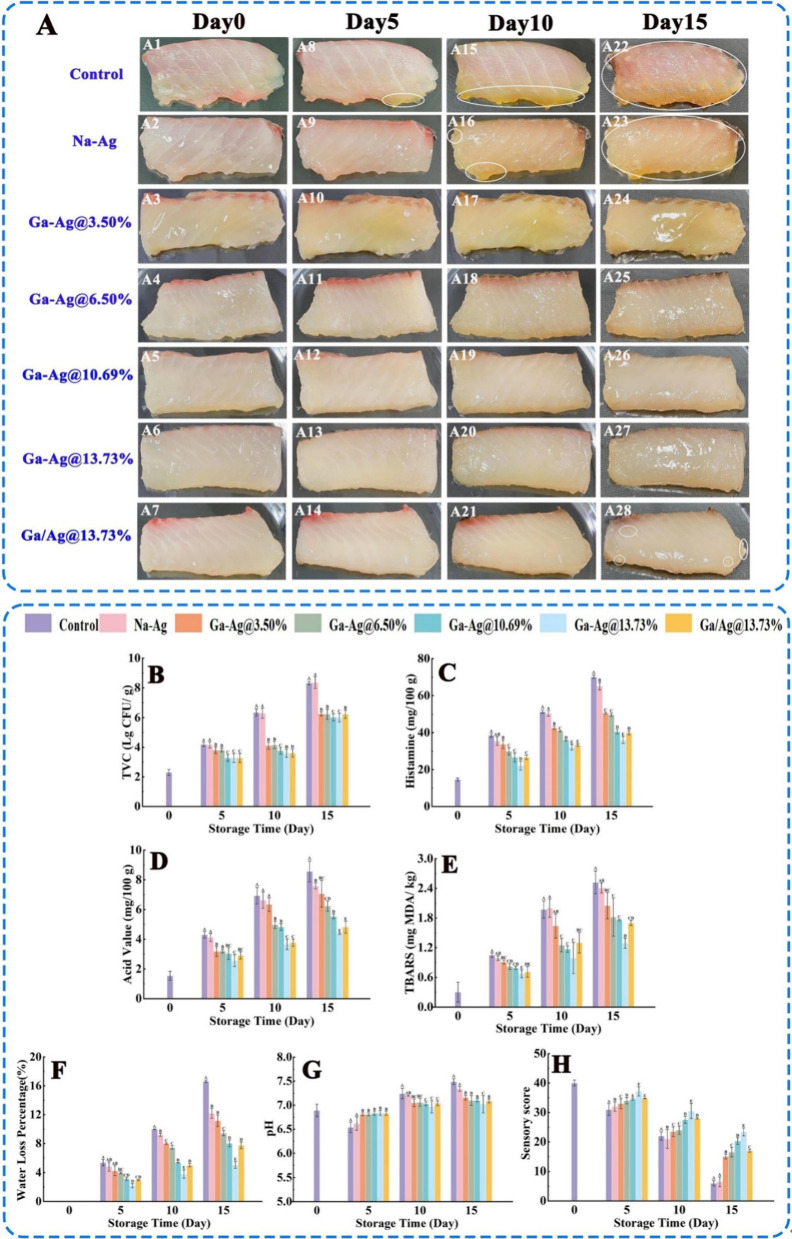
(A) Visual evaluation of grass carp meat coated with sterile water, agarose, modified agarose, and gallic acid/agarose blend. (B) Total viable count (TVC), (C) histamine content, (D) acid value, (E) TBA (thiobarbituric acid) value, (F) water loss percentage, (G) pH value, and (H) sensory scores of grass carp throughout storage. Different uppercase letters indicate statistical significance at *p* < 0.05. Adapted with permission from ref (78). Copyright 2023 Elsevier.

Grass carp fillets were stored at 4 °C for 15 days for study of the preservation capacity (Figure 11). It was observed that, on the 10th day fish coated with control, Na-Ag became yellow and emitted a pungent odor, whereas the fish coated with Ga/Ag blend had a shiny appearance. By the 15th day, fish in the GA/Ag blend had a normal appearance while the fish in control and Na-Ag emitted a pungent odor. The pH of fish was measured, showing that Ga-Ag coatings effectively inhibit microbial growth, enhance water retention, and reduce pH fluctuations, indicating better freshness preservation compared with the control and Na-Ag groups. Initially, the pH decreased due to organic acid formation and then increased after day 5 due to protein breakdown. The TVC was studied (limit, 6–7 log CFU/g), and on the 10th day, control, Na-Ag, Ga-Ag10.69%, and Ga-Ag13.73% exhibited 6.35, 6.30, 6.02, and 6.0 log CFU/g, respectively, indicating that microbial growth is inhibited by Ga/Ag blend coating. Histamine (HIS) is produced in fish on the breaking down of histidine by histidine decarboxylase when fish becomes infected by histamine-producing bacteria. On measuring the HIS content in fish, it was clear that, on the 10th day, control and Na-Ag showed 51.40 and 50.50 mg/100 g, respectively, whereas Ga-Ag@3.50%, Ga-Ag@6.50%, Ga-Ag@10.69%, and Ga-Ag@13.73% exhibited 42.67, 40.79, 35.82, 32.13, and 33.28 mg/100 g HIS content, respectively. This indicated lower growth of histamine-producing bacteria by Ga-Ag blend coatings. From studies, it was clear that Ga-Ag retained the quality of the fillet by inhibiting bacterial growth and fat oxidation, blocking light, and reducing water loss. In this study, though they were able to preserve fish for a longer period, the main disadvantage is the cost of production of modified agarose. Also, the toxicological parameters are to be evaluated before further use.^78^

Zheng and colleagues in 2023^80^ synthesized collagen/gallic acid grafted chitosan/ε-polylysine (CCG/PL) films for pork preservation. ε-Polylysine (2.5, 5, and 10 wt %) was added to prepare films of various ε-polylysine concentrations. Collagen/gallic acid grafted chitosan (CCG) film had higher water solubility than the collagen/chitosan film. The WVPs of CCG/PL-2.5, CCG/PL-5, and CCG/PL-10 films were 3.00 × 10^–12^, 3.01 × 10^–12^, and 3.37 × 10^–12^ g·m^–1^·s^–1^·Pa^–1^, respectively, whereas CC film exhibited 3.16 × 10^–12^ g·m^–1^·s^–1^·Pa^–1^ and CCG exhibited 2.76 × 10^–12^ g·m^–1^·s^–1^·Pa^–1^ WVPs. The CCG films exhibited lower WVP values than CC because of the addition of GA since the availability of hydrophilic amino groups to bind with water molecules was reduced. The GA binds on the −NH_2_ sites in chitosan. Also, the presence of benzene rings in GA, which has hydrophobic behavior, inhibited water passage. In the case of CCG/PL films, due to the hydrophilic nature of ε-polylysine, the WVP increases. This WVP rate points out its disadvantage as a food packaging film. On grafting GA into chitosan, the yellowness of the film was increased whereas the brightness was reduced. However, PL did not affect the color of the film. The TS of CC increases on the addition of GA from 70.94 to 74.26 MPa, and as the PL content increased from 2.5 to 10 wt %, the TS values dropped from 75.15 to 53.24 MPa, while the elongation at break values increased from 35.75 to 42.69%. Similarly, *T*_d_, indicating the denaturation temperature of collagen obtained from DSC, revealed that GA increases the thermal stability of CC, and on incorporation of PL the thermal stability decreases. CC, CCG, CCG/PL-2.5, CCG/PL-5, and CCG/PL-10 had *T*_d_ values of 72.5, 74.4, 74.1, 74.5, and 70.2 °C, respectively. In the case of the UV–vis barrier property on increasing the ε-polylysine concentration, the light transmittance ability was retarded, and this arises due to unsaturated bonds in ε-polylysine which can absorb UV light. The antioxidant activity of the CCG/PL film was higher compared to those of CCG films and CC films. The DDPH radical scavenging activities of CC, CCG, CCG/PL-2.5, CCG/PL-5, and CCG/PL-10 were 3.47, 66.34, 77.17, 81.85, and 87.82%, respectively. These results indicated that antioxidant activity was increased due to the synergistic effect of GA and PL.^79^

**Figure 12:**
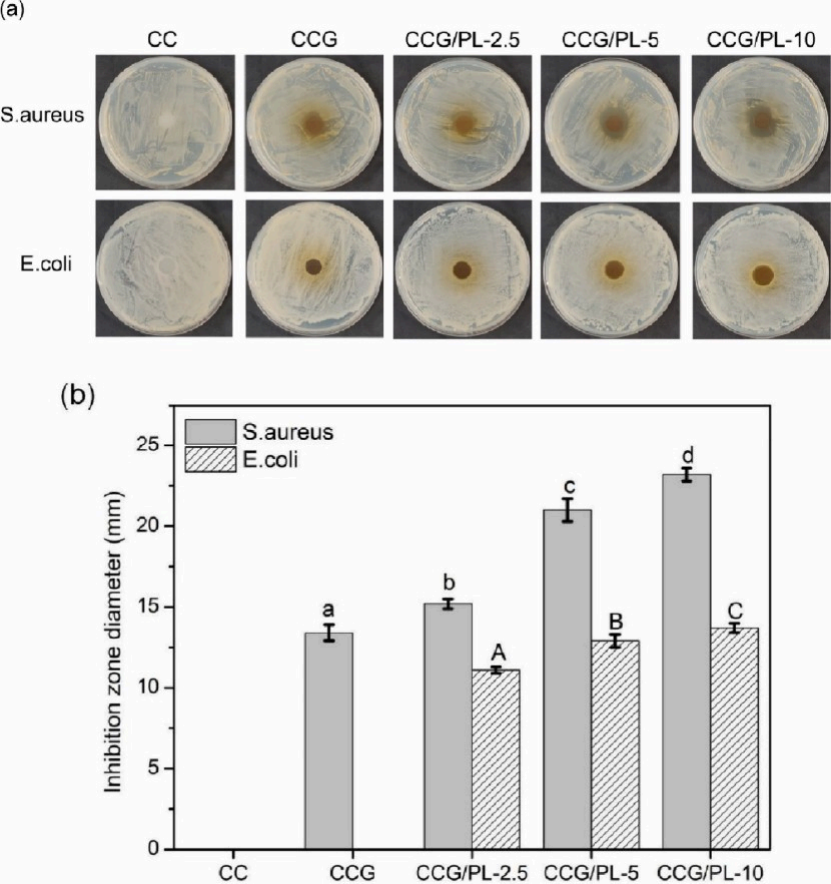
Antimicrobial activities of films against *S. aureus* and *E. coli*. Adapted with permission from ref (80). Copyright 2023 Elsevier.

The antimicrobial activity against *S. aureus* and *E. coli* was tested (Figure 12). CC films showed no inhibition zone but prevented bacterial growth due to chitosan’s antimicrobial properties. CCG films exhibited higher antimicrobial activity after GA addition due to GA’s inherent antimicrobial nature. The inhibition zone diameters for *S. aureus* were 15.2 (CCG/PL-2.5), 21 (CCG/PL-5), and 23.2 mm (CCG/PL-10), while for *E. coli* they were 11.1, 12.9, and 13.7 mm, respectively. This activity results from the interaction between ε-polylysine’s amino groups and the bacterial cell’s negatively charged components, causing cell lysis. The films were tested for pork preservation. The TVBN was studied. It was noted that CCG/PL-5 film showed a TVBN value of 13.63 mg/100 g on the ninth day, whereas the CC film exhibited a higher value than the upper limit (limit, 15 mg/100 g). Similarly, the TBARS value indicating lipid oxidation in pork was calculated (limit, 0.6 mg of MDA/kg). TBARS values of 0.61 and 0.57 mg of MDA/kg were exhibited by the control and CC groups on the sixth day, respectively. However, on the sixth day, the TBARS measurement for pork in the CCG/PL-5 film indicated only 0.35 mg of MDA/kg. This indicated that CCG/PL-5 film excellently reduces lipid oxidation. TVC is another crucial parameter for assessing pork freshness, directly indicating the antibacterial efficacy of the films in preserving pork (limit, 6.00 log CFU/g). Initially, it was 3.12 log CFU/g and for pork preserved as control and CC. The TVC value exceeded the limit on the sixth and ninth days, whereas 5.29 log CFU/g TVC was exhibited on the ninth day and it exceeded the limit on the 12th day. TVBN, TBARS, and TVC demonstrated the excellent efficiency of the film for pork preservation. These results indicate the importance of the film in the upcoming food packaging industry.^80^ A comparison has been made for all of the reported papers in Table 4.

**Table 4:** Comparison of Gallic Acid Based Films for Food Packaging Applications Based on Its Properties

author and year	composition	technique	food preserved	properties enhanced due to GA	ref
Liu et al., 2019	GA-*g*-CS	carbodiimide-mediated coupling reaction, solution casting	white button mushroom (*A. bisporus*)	lowered respiration rate, lower MDA content, higher SOD and CAT, lower PPO, lower ROS	(29)
Aydogdu et al., 2019	PEO/lentil flour/GA	electrospinning	walnut	antioxidant activity, lower peroxide value, lower TOTOX value	(1)
Zhang et al., 2022	CS-GA	coating, photoirradiation	strawberry	antibacterial activity against *E. coli,* pH, and total solid content were stable	(26)
Almeida et al., 2023	TPS/BNC/GA	solvent casting	apple	TS and YM increased, moisture absorption reduced, UV blocking, antioxidant, antibacterial against *S. aureus*, reduction in pH of apple	(46)
Taechutrakul et al., 2023	mPLA-GA	melt processing, hydraulic pressing	banana (*Musa* AAA group)	antioxidant activity, increased oxygen scavenging	(32)
Fang et al., 2018	CS/GA	coating, MAP	pork	antioxidant activity, reduction in TVC, lower TBARS	(51)
Cao et al., 2019	CS/N/GA	coating, HOMAP	pork	reduced pork discoloration, lower TVC, lower TBARS	(54)
Wong et al., 2020	CS/GA	coating, plasma treatment	tilapia (*O. niloticus*)	antioxidant activity, lower TVBN	(33)
Song et al., 2021	COL/ZN/GA	electrospinning	tilapia (*O. niloticus*)	antioxidant activity, film with GA8, lower pH, prolonged meat hardness, lower TVBN, lower TVC	(31)
Xiong et al., 2021	GE-CS-GA-CO	coating	salmon	lowered pH, lower TBARS, lower protein oxidation,	(17)
Huang et al., 2021	GA-Pe and Pr-Pe	enzymatic modification	bass (*L. maculatus*)	lower MDA level, lower acid value	(66)
Ji et al., 2022	YS/CS	solution casting	pork	lowered pH of meat	(81)
Khodanazary et al., 2023	ALG-GA	coating	mackerel (*Sc. commerson*)	reduced histamine content, lower MDA, lower TBA	(73)
Tang et al., 2023	COL/L/GA	solution casting	beef	lower moisture content, lower water solubility, lower water vapor permeability, antioxidant activity, increased UV resistance, increased thermal stability, increased TS, decline in elongation at break, antibacterial activity against *S. aureus* and *E. coli*, COL/L/GA8, lower pH, lower TVC, TVBN, and TBARS	(82)
Fu et al., 2023	Ga-Ag	carbodiimide coupling, coating	grass carp (*C. idellus*)	lower gelling and melting temperatures, antioxidant activity, lower TVC, lower histamine	(78)
Zheng et al., 2023	CCG/PL	solution casting	pork	antioxidant activity, antibacterial, lower WVP, increased thermal stability, lower TVC, TBARS, and TVBN	(80)

## 5 Conclusion

The research findings collectively demonstrate the remarkable potential of gallic acid (GA) as a functional component in the development of active food packaging materials and edible coatings. By incorporating GA into biopolymeric matrixes such as chitosan, poly(ethylene oxide) (PEO), lentil flour, thermoplastic starch (TPS), poly(lactic acid) (PLA), collagen, gelatin, pectin, and agarose, researchers have successfully developed innovative packaging systems with excellent antioxidant, antimicrobial, and preservative properties. These GA-based active packagings have proved effective in extending the shelf life of a wide range of food and meat products, including mushrooms, walnuts, strawberries, fresh-cut apples, bananas, fish, beef, and pork. Due to the presence of GA, these packagings inhibited lipid oxidation, enzymatic browning, and microbial contamination and retarded ripening processes, thereby enhancing food quality, safety, and longevity. The synergistic effects arising from the combination of GA with biopolymers such as chitosan, poly(lactic acid), and bacterial nanocellulose have led to improved mechanical, barrier, and functional properties of the packaging materials.

Optimization of GA loading concentration, exploring wider applications across diverse food products, investigating long-term stability and environmental impact, and conducting comprehensive sensory evaluations and consumer acceptance studies must be taken into account.

Future perspectives in this field include exploring the potential of incorporating GA into edible coatings or films for direct application on food surfaces, which could offer additional preservation benefits. Additionally, researchers could explore the potential of combining GA with other natural antioxidants or antimicrobial agents, potentially leading to synergistic effects and improved overall performance of active packaging systems. Developing cost-effective and scalable production methods for these GA-based packaging materials is also a key area for future research, facilitating their widespread adoption in the food industry.

Overall, the research findings highlight the promising potential of gallic acid as a functional component in active food packaging materials, contributing to improved food quality, safety, and extended shelf life while also encouraging further research and development efforts to overcome existing challenges and facilitate their commercial adoption in the food industry, ultimately benefiting consumers and promoting sustainable food production practices.

## Data Availability

Data will be made available on request.

## Abbreviations

GA: gallic acid
PLA: poly(lactic acid)
PVA: poly(vinyl alcohol)
PE: polyethylene
CS: chitosan
MDA: malondialdehyde
SOD: superoxide dismutase
CAT: catalase
PPO: polyphenol oxidase
PUFA: polyunsaturated fatty acid
TPS: thermoplastic starch
TBARS: thiobarbituric acid reactive substances assay
BNC: bacterial nanocellulose
DPPH: 2,2-diphenyl-1-picrylhydrazyl
l-LA: l-lactide
m-PLA: multibranched PLA
ROP: ring-opening polymerization
PETH: pentaerythritol
ROS: reactive oxygen species
DI: deionized water
RI: ripening index
MAP: modified atmosphere packaging
LAB: lactic acid bacteria
TVC: total viable count
HOMAP: high oxygen modified atmosphere packaging technique
N: nisin
TVBN: total volatile basic nitrogen
OP: oxygen permeability
TPC: total plate count
TBA: thiobarbituric acid
ZN: zein
GE: gelatin
CO: clove oil
Pe: pectin
Pr: propyl gallate
AC: acid value
YS: chinese yam starch
TS: tensile strength
ALG: sodium alginate
BA: biogenic amine
TRY: tryptamine
HIS: histamine
PUT: putrescine
CD: cadaverine
2-PHE: 2-phenylethylamine
TYR: tyramine
SM: spermine
SD: spermidine
COL: collagen
L: laccase
WVP: water vapor permeability
Ag: agarose
WS: water solubility
PL: ε-polylysine
CCG: collagen/gallic acid grafted chitosan
TS: tensile strength
YM: Young’s modulus

## References

[ref1] Aydogdu A.; et al. Enhancing oxidative stability of walnuts by using gallic acid loaded lentil flour based electrospun nanofibers as active packaging material. Food Hydrocoll. 2019, 95, 245–255. 10.1016/j.foodhyd.2019.04.020.

[ref2] V A.; Badwaik L. S. Recent advancement in improvement of properties of polysaccharides and proteins based packaging film with added nanoparticles: A review. Int. J. Biol. Macromol. 2022, 203, 515–525. 10.1016/j.ijbiomac.2022.01.181. 35122798

[ref3] COMA V. Bioactive packaging technologies for extended shelf life of meat-based products. Meat Sci. 2008, 78, 90–103. 10.1016/j.meatsci.2007.07.035. 22062099

[ref4] Díaz-Montes E.; Yáñez-Fernández J.; Castro-Muñoz R. Dextran/chitosan blend film fabrication for bio-packaging of mushrooms (Agaricus bisporus). J. Food Process. Preserv. 2021, 45, e15489 10.1111/jfpp.15489.

[ref5] Jayakumar A.; et al. Recent innovations in bionanocomposites-based food packaging films – A comprehensive review. Food Packag. Shelf Life 2022, 33, 100877 10.1016/j.fpsl.2022.100877.

[ref6] Zhang W.; Rhim J. W. Recent progress in konjac glucomannan-based active food packaging films and property enhancement strategies. Food Hydrocoll. 2022, 128, 107572 10.1016/j.foodhyd.2022.107572.

[ref7] Dharini V.; Periyar Selvam S.; Jayaramudu J.; Sadiku Emmanuel R. Functional properties of clay nanofillers used in the biopolymer-based composite films for active food packaging applications - Review. Appl. Clay Sci. 2022, 226, 106555 10.1016/j.clay.2022.106555.

[ref8] Zhao Y.; et al. Electrospun natural polypeptides based nanofabrics enriched with antioxidant polyphenols for active food preservation. Food Chem. 2023, 405, 134991 10.1016/j.foodchem.2022.134991. 36435113

[ref9] Singh J.; Rai G. K.; Upadhyay A. K.; Kumar R.; Singh K. P. Antioxidant phytochemicals in tomato (Lycopersicon esculentum). Indian J. Agric. Sci. 2004, 74, 3–5.

[ref10] Ma J.; et al. Bioactive Novel Polyphenols from the Fruit of Manilkara zapota (Sapodilla) 2003, 66, 983–986. 10.1021/np020576x. 12880319

[ref11] Shahrzad S.; Bitsch I. Determination of some pharmacologically active phenolic acids in juices by high-performance liquid chromatography 1996, 741, 223–231. 10.1016/0021-9673(96)00169-0. 8785003

[ref12] Eyles B. A.; Davies N. W.; Mitsunaga T.; Mihara R.; Mohammed C. Role of Eucalyptus globulus wound wood extractives. evidence of superoxide dismutase-like activity 2004, 34, 225–232. 10.1111/j.1439-0329.2004.00361.x.

[ref13] Eyles A.; Davies N. W.; Mitsunaga T.; Mihara R.; Mohammed C. Role of Eucalyptus globulus wound wood extractives: Evidence of superoxide dismutase-like activity. For. Pathol. 2004, 34, 225–232. 10.1111/j.1439-0329.2004.00361.x.

[ref14] Subramanian A. P.; et al. Gallic acid: Prospects and molecular mechanisms of its anticancer activity. RSC Adv. 2015, 5, 35608–35621. 10.1039/C5RA02727F.

[ref15] Choubey S.; Varughese L. R.; Kumar V.; Beniwal V. Medicinal importance of gallic acid and its ester derivatives: a patent review. Pharm. Pat. Anal. 2015, 4, 305–315. 10.4155/ppa.15.14. 26174568

[ref16] Wianowska D.; Olszowy-Tomczyk M. A Concise Profile of Gallic Acid—From Its Natural Sources through Biological Properties and Chemical Methods of Determination. Molecules 2023, 28, 1186 10.3390/molecules28031186. 36770851 PMC9919014

[ref17] Xiong Y.; Kamboj M.; Ajlouni S.; Fang Z. Incorporation of salmon bone gelatine with chitosan, gallic acid and clove oil as edible coating for the cold storage of fresh salmon fillet. Food Control 2021, 125, 107994 10.1016/j.foodcont.2021.107994.

[ref18] Cossu A.; et al. Antimicrobial effect of synergistic interaction between UV-A light and gallic acid against Escherichia coli O157:H7 in fresh produce wash water and biofilm. Innov. Food Sci. Emerg. Technol. 2016, 37, 44–52. 10.1016/j.ifset.2016.07.020.

[ref19] Campelo J. E. S.; et al. Evaluation of the acute toxicity of ellagic acid and gallic acid incorporated in Poloxamer407® gel, in Zophobas morio larvae. Toxicol. Vitr. 2024, 95, 105727 10.1016/j.tiv.2023.105727. 37993026

[ref20] Niho N.; et al. Subchronic toxicity study of gallic acid by oral administration in F344 rats. Food Chem. Toxicol. 2001, 39, 1063–1070. 10.1016/S0278-6915(01)00054-0. 11527565

[ref21] Variya B. C.; Bakrania A. K.; Madan P.; Patel S. S. Acute and 28-days repeated dose sub-acute toxicity study of gallic acid in albino mice. Regul. Toxicol. Pharmacol. 2019, 101, 71–78. 10.1016/j.yrtph.2018.11.010. 30465803

[ref22] Stanley J.; et al. Active Agents Incorporated in Polymeric Substrates to Enhance Antibacterial and Antioxidant Properties in Food Packaging Applications. Macromol. 2023, 3, 1–27. 10.3390/macromol3010001.

[ref24] Friedman M.; Jürgens H. S. Effect of pH on the Stability of Plant Phenolic Compounds. J. Agric. Food Chem. 2000, 48, 2101–2110. 10.1021/jf990489j. 10888506

[ref25] Reckziegel P.; et al. Antioxidant protection of gallic acid against toxicity induced by Pb in blood, liver and kidney of rats. Toxicol. Reports 2016, 3, 351–356. 10.1016/j.toxrep.2016.02.005. PMC5615824 28959556

[ref26] Zhang H.; Montemayor A. M.; Wimsatt S. T.; Tikekar R. V. Effect of combination of UV-A light and chitosan-gallic acid coating on microbial safety and quality of fresh strawberries. Food Control 2022, 140, 109106 10.1016/j.foodcont.2022.109106.

[ref27] Campos F. M.; et al. Cell membrane damage induced by phenolic acids on wine lactic acid bacteria. Int. J. Food Microbiol. 2009, 135, 144–151. 10.1016/j.ijfoodmicro.2009.07.031. 19733929

[ref28] Zhang X.; Liu J.; Qian C.; Kan J.; Jin C. Effect of grafting method on the physical property and antioxidant potential of chitosan film functionalized with gallic acid. Food Hydrocoll. 2019, 89, 1–10. 10.1016/j.foodhyd.2018.10.023.

[ref29] Liu J.; Liu S.; Zhang X.; Kan J.; Jin C. Effect of gallic acid grafted chitosan film packaging on the postharvest quality of white button mushroom (Agaricus bisporus). Postharvest Biol. Technol. 2019, 147, 39–47. 10.1016/j.postharvbio.2018.09.004.

[ref30] Nájera-Martínez E. F.; et al. Microencapsulation of Gallic Acid Based on a Polymeric and pH-Sensitive Matrix of Pectin/Alginate. Polymers (Basel) 2023, 15, 3014 10.3390/polym15143014. 37514404 PMC10384038

[ref31] Song Z.; et al. Collagen/zein electrospun films incorporated with gallic acid for tilapia (Oreochromis niloticus) muscle preservation. J. Food Eng. 2022, 317, 110860 10.1016/j.jfoodeng.2021.110860.

[ref32] Taechutrakul S.; Piroonpan T.; Pasanphan W. Active film strips to extend the shelf life of fruits: Multibranched PLA-gallic acid as an antioxidant/oxygen scavenger in a case study of bananas (Musa AAA group). J. Food Eng. 2024, 364, 111794 10.1016/j.jfoodeng.2023.111794.

[ref33] Wong L.-W.; et al. Use of the plasma-treated and chitosan/gallic acid-coated polyethylene film for the preservation of tilapia (Orechromis niloticus) fillets. Food Chem. 2020, 329, 126989 10.1016/j.foodchem.2020.126989. 32502742

[ref34] Promsorn J.; Harnkarnsujarit N. Oxygen absorbing food packaging made by extrusion compounding of thermoplastic cassava starch with gallic acid. Food Control 2022, 142, 109273 10.1016/j.foodcont.2022.109273.

[ref35] Mahajan P. V.; Rodrigues F. A. S.; Motel A.; Leonhard A. Development of a moisture absorber for packaging of fresh mushrooms (Agaricus bisporous). Postharvest Biol. Technol. 2008, 48, 408–414. 10.1016/j.postharvbio.2007.11.007.

[ref36] Ahn B. J.; Gaikwad K. K.; Lee Y. S. Characterization and properties of LDPE film with gallic-acid-based oxygen scavenging system useful as a functional packaging material. J. Appl. Polym. Sci. 2016, 133, 44138 10.1002/app.44138.

[ref37] Wang Z.; Yang L. Delinking indicators on regional industry development and carbon emissions: Beijing–Tianjin–Hebei economic band case. Ecol. Indic. 2015, 48, 41–48. 10.1016/j.ecolind.2014.07.035.

[ref38] Hu Y.-H.; et al. Postharvest application of 4-methoxy cinnamic acid for extending the shelf life of mushroom (Agaricus bisporus). Postharvest Biol. Technol. 2015, 104, 33–41. 10.1016/j.postharvbio.2015.03.007.

[ref39] Meng D.; Song T.; Shen L.; Zhang X.; Sheng J. Postharvest Application of Methyl Jasmonate for Improving Quality Retention of Agaricus bisporus Fruit Bodies. J. Agric. Food Chem. 2012, 60, 6056–6062. 10.1021/jf3006454. 22657158

[ref40] da Rosa C. G.; et al. Microencapsulation of gallic acid in chitosan, β-cyclodextrin and xanthan. Ind. Crops Prod. 2013, 46, 138–146. 10.1016/j.indcrop.2012.12.053.

[ref41] Arrieta M. P.; Peltzer M. A.; Garrigós M. D. C.; Jiménez A. Structure and mechanical properties of sodium and calcium caseinate edible active films with carvacrol. J. Food Eng. 2013, 114, 486–494. 10.1016/j.jfoodeng.2012.09.002.

[ref42] Zhang Y.; et al. Oxidative stability of sunflower oil supplemented with carnosic acid compared with synthetic antioxidants during accelerated storage. Food Chem. 2010, 118, 656–662. 10.1016/j.foodchem.2009.05.038. 25214334

[ref43] Karimi S.; Tahir P. Md.; Dufresne A.; Karimi A.; Abdulkhani A. A comparative study on characteristics of nanocellulose reinforced thermoplastic starch biofilms prepared with different techniques. Nord. Pulp Pap. Res. J. 2014, 29, 41–45. 10.3183/npprj-2014-29-01-p041-045.

[ref44] Mangmee K.; Homthawornchoo W. Antioxidant activity and physicochemical properties of rice starch-chitosan-based films containing green tea extract. Food Appl. Biosci. J. 2016, 4, 126–137. 10.14456/fabj.2016.12.

[ref45] Borges A.; Ferreira C.; Saavedra M. J.; Simões M. Antibacterial Activity and Mode of Action of Ferulic and Gallic Acids Against Pathogenic Bacteria. Microb. Drug Resist. 2013, 19, 256–265. 10.1089/mdr.2012.0244. 23480526

[ref46] Almeida T.; et al. Biobased ternary films of thermoplastic starch, bacterial nanocellulose and gallic acid for active food packaging. Food Hydrocoll. 2023, 144, 108934 10.1016/j.foodhyd.2023.108934.

[ref47] Petrou S.; Tsiraki M.; Giatrakou V.; Savvaidis I. N. Chitosan dipping or oregano oil treatments, singly or combined on modified atmosphere packaged chicken breast meat. Int. J. Food Microbiol. 2012, 156, 264–271. 10.1016/j.ijfoodmicro.2012.04.002. 22534355

[ref48] Fan W.; et al. Effects of chitosan coating on quality and shelf life of silver carp during frozen storage. Food Chem. 2009, 115, 66–70. 10.1016/j.foodchem.2008.11.060.

[ref49] Sun X.; Wang Z.; Kadouh H.; Zhou K. The antimicrobial, mechanical, physical and structural properties of chitosan-gallic acid films. Lwt 2014, 57, 83–89. 10.1016/j.lwt.2013.11.037.

[ref50] Badhani B.; Sharma N.; Kakkar R. Gallic acid: a versatile antioxidant with promising therapeutic and industrial applications. RSC Adv. 2015, 5, 27540–27557. 10.1039/C5RA01911G.

[ref51] Fang Z.; Lin D.; Warner R. D.; Ha M. Effect of gallic acid/chitosan coating on fresh pork quality in modified atmosphere packaging. Food Chem. 2018, 260, 90–96. 10.1016/j.foodchem.2018.04.005. 29699687

[ref52] Samelis J.; Georgiadou K. G. The microbial association of Greek taverna sausage stored at 4 and 10 °C in air, vacuum or 100% carbon dioxide, and its spoilage potential. J. Appl. Microbiol. 2000, 88, 58–68. 10.1046/j.1365-2672.2000.00936.x. 10735244

[ref53] Huang X.; et al. Determination of pork spoilage by colorimetric gas sensor array based on natural pigments. Food Chem. 2014, 145, 549–554. 10.1016/j.foodchem.2013.08.101. 24128513

[ref54] Cao Y.; Warner R. D.; Fang Z. Effect of chitosan/nisin/gallic acid coating on preservation of pork loin in high oxygen modified atmosphere packaging. Food Control 2019, 101, 9–16. 10.1016/j.foodcont.2019.02.013.

[ref55] Remya S.; et al. Effect of chitosan based active packaging film on the keeping quality of chilled stored barracuda fish. J. Food Sci. Technol. 2016, 53, 685–693. 10.1007/s13197-015-2018-6. 26787988 PMC4711446

[ref56] Hui G.; Liu W.; Feng H.; Li J.; Gao Y. Effects of chitosan combined with nisin treatment on storage quality of large yellow croaker (Pseudosciaena crocea). Food Chem. 2016, 203, 276–282. 10.1016/j.foodchem.2016.01.122. 26948615

[ref57] Xin S.; et al. Preparation of chitosan/curcumin nanoparticles based zein and potato starch composite films for Schizothorax prenati fillet preservation. Int. J. Biol. Macromol. 2020, 164, 211–221. 10.1016/j.ijbiomac.2020.07.082. 32679329

[ref58] Burcham P. C.; Kuhan Y. T. Introduction of Carbonyl Groups into Proteins by the Lipid Peroxidation Product, Malondialdehyde. Biochem. Biophys. Res. Commun. 1996, 220, 996–1001. 10.1006/bbrc.1996.0521. 8607882

[ref59] Varma A. J.; Deshpande S. V.; Kennedy J. F. Metal complexation by chitosan and its derivatives: A review. Carbohydr. Polym. 2004, 55, 77–93. 10.1016/j.carbpol.2003.08.005.

[ref60] Park P. J.; Je J. Y.; Kim S. K. Free radical scavenging activities of differently deacetylated chitosans using an ESR spectrometer. Carbohydr. Polym. 2004, 55, 17–22. 10.1016/j.carbpol.2003.05.002.

[ref61] Xiong Y.; Chen M.; Warner R. D.; Fang Z. Incorporating nisin and grape seed extract in chitosan-gelatine edible coating and its effect on cold storage of fresh pork. Food Control 2020, 110, 107018 10.1016/j.foodcont.2019.107018.

[ref62] Yu D.; Regenstein J. M.; Xia W. Bio-based edible coatings for the preservation of fishery products: A Review. Crit. Rev. Food Sci. Nutr. 2019, 59, 2481–2493. 10.1080/10408398.2018.1457623. 29584448

[ref63] Rui L.; et al. A comparative study on chitosan/gelatin composite films with conjugated or incorporated gallic acid. Carbohydr. Polym. 2017, 173, 473–481. 10.1016/j.carbpol.2017.05.072. 28732889

[ref64] Cortés-Rojas D. F.; de Souza C. R. F.; Oliveira W. P. Clove (Syzygium aromaticum): a precious spice. Asian Pac. J. Trop. Biomed. 2014, 4, 90–96. 10.1016/S2221-1691(14)60215-X. 25182278 PMC3819475

[ref65] Hassani A.; Azarian M. M. S.; Ibrahim W. N.; Hussain S. A. Preparation, characterization and therapeutic properties of gum arabic-stabilized gallic acid nanoparticles. Sci. Rep. 2020, 10, 17808 10.1038/s41598-020-71175-8. 33082415 PMC7576211

[ref66] Huang B.; et al. Investigation of the pectin grafting with gallic acid and propyl gallate and their antioxidant activities, antibacterial activities and fresh keeping performance. Int. J. Biol. Macromol. 2021, 190, 343–350. 10.1016/j.ijbiomac.2021.08.219. 34492247

[ref67] Chi C.; et al. Modulating the in vitro digestibility and predicted glycemic index of rice starch gels by complexation with gallic acid. Food Hydrocoll. 2019, 89, 821–828. 10.1016/j.foodhyd.2018.11.016.

[ref68] Zhao Y.; Teixeira J. S.; Gänzle M. M.; Saldaña M. D. A. Development of antimicrobial films based on cassava starch, chitosan and gallic acid using subcritical water technology. J. Supercrit. Fluids 2018, 137, 101–110. 10.1016/j.supflu.2018.03.010.

[ref69] Rong L.; et al. Characterization of gallic acid-Chinese yam starch biodegradable film incorporated with chitosan for potential use in pork preservation. Food Res. Int. 2023, 164, 112331 10.1016/j.foodres.2022.112331. 36737924

[ref70] Fish and Fishery Products Hazards and Controls Guidance, 4th ed.; FDA: 2011.

[ref71] Ruan C.; et al. Preparation and antioxidant activity of sodium alginate and carboxymethyl cellulose edible films with epigallocatechin gallate. Int. J. Biol. Macromol. 2019, 134, 1038–1044. 10.1016/j.ijbiomac.2019.05.143. 31128181

[ref72] Zarandona I.; et al. Horse mackerel (Trachurus trachurus) fillets biopreservation by using gallic acid and chitosan coatings. Food Control 2021, 120, 107511 10.1016/j.foodcont.2020.107511.

[ref73] Khodanazary A.; Mohammadzadeh B. Effect of alginate-gallic acid coating on freshness and flavor properties of Mackerel (Scomberomorus commerson) fillets under refrigerated storage. Int. J. Biol. Macromol. 2023, 249, 125999 10.1016/j.ijbiomac.2023.125999. 37499710

[ref74] Mittal A.; et al. Composite films based on chitosan and epigallocatechin gallate grafted chitosan: Characterization, antioxidant and antimicrobial activities. Food Hydrocoll. 2021, 111, 106384 10.1016/j.foodhyd.2020.106384.

[ref75] Alves M. J.; et al. Antimicrobial activity of phenolic compounds identified in wild mushrooms, SAR analysis and docking studies. J. Appl. Microbiol. 2013, 115, 346–357. 10.1111/jam.12196. 23510516

[ref76] Tang P.; Zheng T.; Yang C.; Li G. Enhanced physicochemical and functional properties of collagen films cross-linked with laccase oxidized phenolic acids for active edible food packaging. Food Chem. 2022, 393, 133353 10.1016/j.foodchem.2022.133353. 35679702

[ref77] Ghosh A. K.; Shahabi D. Synthesis of amide derivatives for electron deficient amines and functionalized carboxylic acids using EDC and DMAP and a catalytic amount of HOBt as the coupling reagents. Tetrahedron Lett. 2021, 63, 152719 10.1016/j.tetlet.2020.152719. 33456089 PMC7808253

[ref78] Fu L.; et al. Bio-based active packaging: Gallic acid modified agarose coatings in grass carp (Ctenopharyngodon idellus) preservation. Int. J. Biol. Macromol. 2024, 255, 128196 10.1016/j.ijbiomac.2023.128196. 37984583

[ref79] Li Q.; et al. Effects of gallic acid combined with epsilon-polylysine hydrochloride incorporated in a pullulan–CMC edible coating on the storage quality of sea bass. RSC Adv. 2021, 11, 29675–29683. 10.1039/D1RA02320A. 35479553 PMC9040880

[ref80] Zheng T.; Tang P.; Yang C.; Ran R.; Li G. Development of active packaging films based on collagen/gallic acid-grafted chitosan incorporating with ε-polylysine for pork preservation. Food Hydrocoll. 2023, 140, 108590 10.1016/j.foodhyd.2023.108590.

[ref81] Ji X.; et al. Gallic acid and heat moisture treatment improve pasting, rheological, and microstructure properties of Chinese yam starch-chitosan gels: A comparative study. Int. J. Biol. Macromol. 2022, 222, 114–120. 10.1016/j.ijbiomac.2022.09.090. 36113602

[ref82] Tang P.; Zheng T.; Ran R.; Xiong Y.; Li G. Collagen films functionalized with gallic acid in the presence of laccase for beef preservation. Food Packag. Shelf Life 2023, 38, 101100 10.1016/j.fpsl.2023.101100.

